# Systematic review of the evidence relating FEV_1 _decline to giving up smoking

**DOI:** 10.1186/1741-7015-8-84

**Published:** 2010-12-14

**Authors:** Peter N Lee, John S Fry

**Affiliations:** 1 P.N. Lee Statistics and Computing Ltd, Surrey, UK

## Abstract

**Background:**

The rate of forced expiratory volume in 1 second (FEV_1_) decline ("beta") is a marker of chronic obstructive pulmonary disease risk. The reduction in beta after quitting smoking is an upper limit for the reduction achievable from switching to novel nicotine delivery products. We review available evidence to estimate this reduction and quantify the relationship of smoking to beta.

**Methods:**

Studies were identified, in healthy individuals or patients with respiratory disease, that provided data on beta over at least 2 years of follow-up, separately for those who gave up smoking and other smoking groups. Publications to June 2010 were considered. Independent beta estimates were derived for four main smoking groups: never smokers, ex-smokers (before baseline), quitters (during follow-up) and continuing smokers. Unweighted and inverse variance-weighted regression analyses compared betas in the smoking groups, and in continuing smokers by amount smoked, and estimated whether beta or beta differences between smoking groups varied by age, sex and other factors.

**Results:**

Forty-seven studies had relevant data, 28 for both sexes and 19 for males. Sixteen studies started before 1970. Mean follow-up was 11 years. On the basis of weighted analysis of 303 betas for the four smoking groups, never smokers had a beta 10.8 mL/yr (95% confidence interval (CI), 8.9 to 12.8) less than continuing smokers. Betas for ex-smokers were 12.4 mL/yr (95% CI, 10.1 to 14.7) less than for continuing smokers, and for quitters, 8.5 mL/yr (95% CI, 5.6 to 11.4) less. These betas were similar to that for never smokers. In continuing smokers, beta increased 0.33 mL/yr per cigarette/day. Beta differences between continuing smokers and those who gave up were greater in patients with respiratory disease or with reduced baseline lung function, but were not clearly related to age or sex.

**Conclusion:**

The available data have numerous limitations, but clearly show that continuing smokers have a beta that is dose-related and over 10 mL/yr greater than in never smokers, ex-smokers or quitters. The greater decline in those with respiratory disease or reduced lung function is consistent with some smokers having a more rapid rate of FEV_1 _decline. These results help in designing studies comparing continuing smokers of conventional cigarettes and switchers to novel products.

## Background

It is generally believed that 15% to 20% of all long-term regular smokers will develop clinically overt chronic obstructive pulmonary disease (COPD) and that most COPD cases worldwide are attributable to cigarette smoking [1]. COPD, along with ischaemic heart disease and lung cancer, is a major contributor to the number of deaths caused by smoking [2]. In this paper, we attempt to provide information relevant to determining the maximum reduction in risk of COPD that might be achieved from the introduction of new-generation nicotine delivery products that are currently being developed with the aim of substantially reducing the risks of tobacco-related disease. There are two underlying assumptions: that any benefits from the introduction of these products cannot exceed those of giving up smoking and that the rate of decline over time in forced expiratory volume in 1 second (FEV_1_) is a reliable marker of the risk of COPD. FEV_1 _is also a marker of other respiratory diseases such as asthma, pulmonary fibrosis or cystic fibrosis. The information we provide should also be relevant in a wider context, such as research into the use of non-nicotine-containing drugs in the area of smoking cessation and more generally in accurately conveying the hazards of smoking.

We present a systematic review of the epidemiological evidence on the relationship of smoking status to the rate of FEV_1 _decline. While our major interest is in the comparison of rates in continuing smokers and those who gave up, we also summarize information on the rate of FEV_1 _decline in never smokers and on the relationship of FEV_1 _decline to amount smoked. We also investigate how differences in the rate of FEV_1 _decline by smoking status are affected by other factors, with the aim of identifying those subgroups that show the largest differences between continuing smokers and those who give up smoking.

It is well known that continuing smokers have an average rate of decline in FEV_1 _that is substantially greater than that of people who have never smoked [2], and a recent review by Willemse *et al. *[3] summarizes some data demonstrating that giving up smoking reduces the rate of decline in smokers without chronic symptoms, in smokers with nonobstructive chronic bronchitis and in smokers with COPD. However, summary estimates, based on all available data, of the extent of the decline in those who give up smoking relative to those who continue to smoke is not available in the literature, and a major aim of our paper is to provide this information.

We restrict attention to studies providing data on FEV_1 _in the same individuals at more than one time point and also data for those who give up smoking. Four smoking groups are particularly relevant to the analyses, and, to avoid confusion, we henceforward consistently describe them as "never smokers", "ex-smokers", "quitters" and "continuing smokers". We define "quitters" as subjects smoking at the start of the follow-up period but not still smoking at the end, and "ex-smokers" as those who had given up smoking by the start of the follow-up period and did not resume smoking during it. "Continuing smokers" are those reporting current smoking at the start and end of the period, and "never smokers" are those reporting never having smoked at both time points. For convenience, we also routinely use the term "beta" to mean the estimated rate of decline of FEV_1 _in millilitres per year over the follow-up period, with a positive beta implying a lower FEV_1 _at the end of the period. Beta is often used in statistical contexts to describe the slope of a line.

## Methods

### Selection of studies

Studies selected had to satisfy five conditions: (1) FEV_1 _must be measured in the same individuals at least twice over a period of at least 2 years; (2) data must be reported separately for those who give up smoking, with randomized studies reporting results only by advice to quit smoking being excluded; (3) results for a quantitative index of FEV_1 _decline over a period must be available directly or calculable from the data presented; (4) subjects studied must be adults (or present results for an age group, such as 15+ years, consisting predominantly of adults); and (5) subjects studied may be healthy individuals or patients with COPD, chronic bronchitis or emphysema, but not patients with other specific conditions (for example, α_1_-antitrypsin deficiency) or workers in occupations with a high risk of disease (for example, miners).

Relevant publications were initially sought from a MedLine search conducted on 6 April 2009, on "(Lung function or FEV_1 _or decline in FEV_1_) and (ex-smokers or smoking cessation)" limited to "Humans" and to "All adults: 19+ years", from publications cited in Table 2 and 3 of a review by Willemse *et al*. in 2004 [3], from the relevant chapter of an International Agency for Research on Cancer handbook in 2007 [1], from an earlier unpublished collection of literature on smoking and FEV_1 _(Alison Thornton, personal communication, 28 October 2004) and from reference lists of papers identified. Subsequently, on 6 July 2010, the search was updated to 30 June 2010, with new publications being identified from this search and from reference lists of papers identified. Fuller details of the search strategy are given in Additional file 1 FEV1 search strategy.doc.

### Data entry

For each study, relevant data were entered into a study database and a beta database. The study database contains a single record for each study describing various study attributes, including relevant publications, sexes considered, age range, location, timing, length of follow-up, study design (prospective or intervention study, study of the general population or of patients with specified respiratory diseases, nature of the population, exclusions, study size and use of bronchodilators for measuring FEV_1_), availability of FEV_1 _results (beta, beta relative to never smokers, beta relative to continuing smokers and other indices such as beta divided by height cubed or FEV_1 _change as a percentage of predicted), potential confounding variables used for one or more betas, availability of results for different aspects of smoking, availability of betas stratified by sex, age and other stratifying variables, and the number of beta records in the beta database.

The beta database contains a record for each beta for each study. This record is divided into four parts. The first part gives the smoking habits at the start and end of the follow-up period classified by smoking status (never smoker, ex-smoker, current smoker, ever smoked and nonsmoker), smoking product (any, cigarettes ± other products, cigarettes only, pipes or cigars only or pipe only), cigarette type (any, manufactured only or hand-rolled only) and, where relevant, details of dose-response variables (measure of exposure and range of values, for example, 10-19 cigarettes/day). The second part of the beta record gives the sex and age of the individuals to whom the beta relates and, where applicable, details of other stratifying variables (such as baseline FEV_1 _level or whether histamine-responsive or not). The third part gives details of potential confounding variables taken into account when estimating the beta. The final part contains the beta data, giving the type of beta (direct, relative to never smokers or relative to continuing smokers), the value itself expressed as the decline in millilitres per year (with negative values indicating an increase) and available information relevant to the variability of the beta (the lower and upper 95% confidence interval (CI), standard deviation (SD), standard error (SE) and number of subjects the beta is based on (*N*)). It also contains information on how the beta was derived and the length of the period studied. Details are also entered for the reference group for betas relative to never smokers (never anything, never cigarettes) and for betas relative to continuing smokers (any product, cigarettes). Commentary also provides further detail relating to the beta where necessary.

It should be noted that the beta database contains only data relevant to betas estimated directly relative to never smokers or relative to continuing smokers. Data for indices such as FEV_1 _decline per year divided by height cubed or percentage change from baseline are available for very few studies, and data on FEV_1 _change as a percentage of predicted, though available for rather more studies, would have been difficult to use in meta-analysis because of the varying definition of the predicted value.

In some studies, the estimate of beta is given directly, but in others it was estimated by dividing the difference between FEV_1 _values given at the start and end of follow-up by the length of follow-up. The length of follow-up itself was not always provided precisely and sometimes had to be estimated from information given on the timing of the relevant surveys. Where necessary, betas and their SDs or SEs were estimated from data given graphically or by individual subject.

Fuller details of the variables recorded in the databases are given in Additional file 2 Data recorded.doc.

### Statistical analysis

Most analyses were carried out on the basis of unweighted and inverse variance-weighted linear multiple regression analysis. For the weighted analysis, an estimate of SE was required. For some betas, the SE was given directly, and in others it could be calculated directly using standard formulae from available information on the 95% CI or on *N *and SD combined. For some betas, information was available on *N*, but not on variability (SD, SE or CI). For those betas, SE was estimated from the age-specific mean SD for those other betas where the SD was directly available. For some betas, none of *N*, SD, SE or CI was provided, so the SE could not be estimated, the beta not being included in the weighted analysis. In principle, the SE could have been estimated from the beta and its associated *P *value. However, *P *values were rarely available, and where provided they were not given to sufficient accuracy (for example, only as *P *< 0.05) to allow reasonable estimation of the SE.

The main analyses were conducted on the four smoking groups already described: never smokers, ex-smokers, quitters and continuing smokers. Estimates of beta were not included in the analysis if information on smoking habits was lacking at the start or end of follow-up if the betas related to other smoking groups (for example, never smokers at the start who smoked during follow-up) or were for smokers of pipes and/or cigars only. While multiple betas for the same smoking group and the same study could be included, provided they were independent (for example, estimates for different sexes, age groups or levels of other stratifying variables), only one beta was chosen from nonindependent alternatives. Where there was a choice, preference was given to betas based on the longest follow-up time, betas given separately by age, betas adjusted for the most variables, betas where the SE was available or could be calculated, betas based on FEV_1 _measurements taken without bronchodilator and for other study-specific reasons described in the Results.

Analyses were carried out using fixed-effects linear regression models to compare the four smoking groups, without adjustment for other variables, with adjustment for both sex (males, females, and sexes combined) and age (midpoint of age interval in the ranges <40, 40-49, 50-69, and 70+), and with adjustment for "block", a block being a set of betas from the same study and for the same levels of stratifying variables. Data within a block are presented on the same row in the tables presenting the beta data used in the analyses. The fixed-effects block-adjusted analyses fit a separate term for each block. The results of an alternative analysis using a random effects model in which block effects were assumed to be normally distributed are also shown.

The relationship of beta to sex, age and various other factors (length of follow-up period, continental location of the study, final follow-up year, publication year, population type and study type) was also studied using weighted and unweighted fixed-effects linear multiple regression analysis based on models including sex, age, smoking group and the factor of interest. Estimates of beta with 95% CI are presented by level of smoking group and factor. Differences between betas for smoking groups are also presented, with the significance of the difference presented as *P *< 0.001, *P *< 0.01, *P *< 0.05 or *P *≥ 0.05. For differences between levels of the factors, only the significance level is presented.

Similar unweighted and inverse variance-weighted linear multiple regression analyses were also carried out based on differences in betas within the same block between (1) continuing smokers and quitters and (2) continuing smokers and ex-smokers. Whereas the analyses involving data from all four smoking groups test whether beta varies by other factors, such as age and sex, these analyses test whether the specified differences in beta between smoking groups varies by these factors. For the purpose of the analysis of differences, the SE of a difference was estimated as the root mean square of the SEs of the two betas concerned.

Analyses were also carried out comparing betas by amount smoked. These were restricted to betas which concerned continuing smokers, where the unit of exposure was cigarettes/day, where the subject stayed in the same exposure group between the start and end of follow-up and where preference was given to estimates adjusted for the most variables. Unweighted and inverse variance-weighted linear regression analyses related beta to cigarettes/day after adjustment for block using fixed-effects modelling. As the data for a given beta were available only for a range of cigarettes/day smoked, the value used in the regression analyses was the mean of the lower and upper limits (for example, mean 19.5 cigarettes/day for 15-24 cigarettes/day). For the highest consumption groups, which are open-ended, the mean was estimated assuming that the upper limit was 50 cigarettes/day (for example, mean 37.5 cigarettes/day for 25+ cigarettes/day).

In interpreting the analyses described above, the most importance was given to the results from the inverse variance-weighted analyses adjusted for block where relevant using fixed-effects modelling. The unadjusted analyses and those adjusted for age and sex may be somewhat biased by the fact that the results for the different smoking groups come from different sets of studies.

Some studies reported betas separately by level of other factors, each as baseline FEV_1_, bronchodilator responsiveness or occupational exposure. To assess whether beta was associated with the factor, the unweighted and inverse variance-weighted trends in beta (increase per level) and their SEs were estimated separately for continuing smokers, quitters, ex-smokers, for the difference between quitters and continuing smokers and for the difference between ex-smokers and continuing smokers.

The heterogeneity of beta estimates was assessed separately for the four smoking groups by an F-test comparing the between-study variance in betas with the within-study variance. To avoid complications due to the SE of beta for some studies having to be calculated indirectly, this assessment was limited to those studies where the SE was provided or could be calculated from the SD and *N *and where *N *was known.

### Software

ROELEE version 3.1 software (available from P.N. Lee Statistics and Computing Ltd., Sutton, Surrey, UK) was used for entry of the data into the study and beta databases and for virtually all the statistical analyses. The data were then transferred to SAS version 9.1 software (SAS Institute Inc., Cary, NC, USA), and the analyses run on ROELEE were rerun as a cross-check. The analyses treating block as a random effect were run only in SAS.

## Results

### The studies

**Figure 1:**
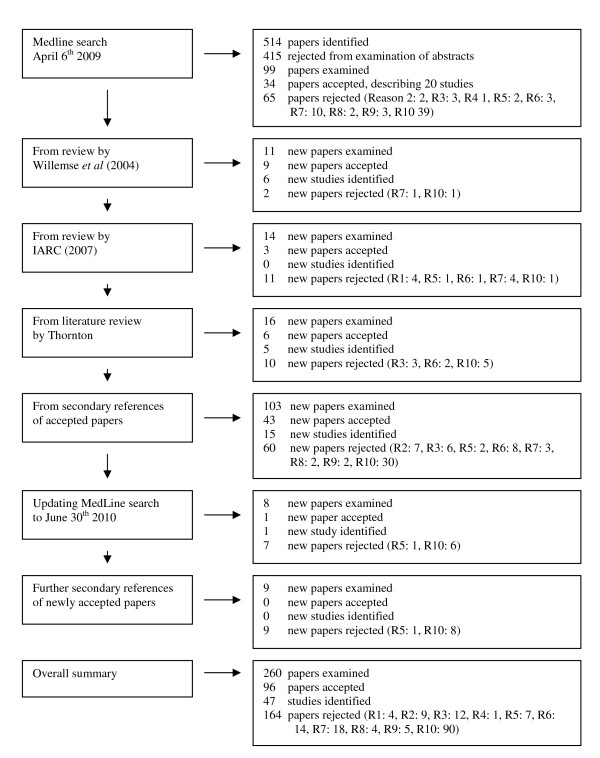
**Flow diagram for literature search**. The diagram shows the number of papers and studies identified, examined, accepted and rejected at the different stages of the literature search. Reasons for rejection of papers are coded as follows. R1, paper unobtainable; R2, study of patients with specified conditions that are not chronic obstructive pulmonary disease; R3, study of workers in high-risk occupations; R4, study of children; R5, review paper with no new studies mentioned; R6, not a prospective study; R7, follow-up period too short (< 2 yr); R8, no follow-up data; R9, data only for FEV_0.75_; R10, no relevant data on FEV_1 _decline in those who gave up smoking. Fuller details are given in Additional file 1 FEV1 search strategy.doc.

From the abstracts of the publications identified in the initial search in 2009 and the update in 2010, it was often possible to tell that no relevant data were available, and after excluding these publications, a total of 260 were examined in detail, with 96 publications finally accepted. These publications related to 47 studies. Additional file 1 FEV_1 _search strategy.doc, gives fuller details of the progress of the search, summarized as a flow diagram in Figure 1. Of the 47 studies finally identified, only 20 were identified directly from the initial MedLine search in 2009, with a further 11 identified from other reviews and 15 from secondary references. The updated search identified only one additional study.

Table 1 summarizes the details of the baseline population, exclusions, location and follow-up period of the 47 studies identified, with studies identified in 2009 numbered 1 to 46 and the study identified in 2010 numbered 47. Table 2 gives a distribution of various study characteristics. Thirty-seven studies can be broadly classified as relating to the general population (though a number of the studies excluded subjects with specified diseases), with six relating to subjects with specified respiratory diseases (or, in the case of study 37, to men with some potential risk factors for FEV_1 _decline). Four were intervention studies, with the presence of a specified respiratory disease being a requirement for three of these (studies 43, 44 and 46). Twenty-eight studies involved both sexes, and the remainder involved males only. Fifteen studies were restricted to relatively young people, with a maximum age at baseline of 59 years, while three studies were restricted to relatively older adults, with a minimum age at baseline of at least 60 years. Of the 44 single-country studies, 13 were conducted in the USA, 2 in Canada, 6 in the UK, 7 in Western Europe, 7 in Scandinavia, 2 in Eastern Europe, 6 in Asia and 1 in Australia. There were also three multicountry studies (studies 7, 43 and 44). Many of the studies started many years ago: 16 began in 1960-1969 and 15 began in 1970-1979. On the basis of the difference between the year of start at baseline and the year of the end of follow-up, the mean length of follow-up is estimated as 11 years, with seven studies taking at least 20 years. An attempt was made to determine, for each study, the number of individuals entering the follow-up period. This ranged from a minimum of 13 individuals in study 38 to 9,317 individuals in study 1. Twenty-one studies included at least 1,000 individuals.

**Table 1:** Details of studies providing data on FEV1 decline in people who gave up smoking^a^

Study **no**.	References^b^	Location	Follow-up period^c^	Baseline population^d^	Exclusions
	General population studies				
					
1	Bartholomew and Knuiman 1998 [12], James *et al*. 2005 [13]	Busselton, Australia	1966-1975 to 1995	9,317 men and women ages 18+ attending health surveys	None
					
2	Beck *et al*. 1982 [14]	Lebanon, CT, USA	1972-1978	632 white men and women, residents of a rural community ages 25+^e^	None
					
3	Bosse *et al*. 1980 [15], Bosse *et al*. 1981 [16], Gottlieb *et al*. 1996 [17], Sparrow *et al*. 1983 [18], Sparrow *et al*. 1984 [19], Sparrow *et al*. 1993 [20]	Boston, MA, USA	1963-1968 to 1978	2,000 male volunteers ages 20-80 (Normative aging study)	Chronic medical conditions
					
4	Burchfiel *et al*. 1995 [11]	Hawaii, USA	1965-1968 to 1975	4,451 Japanese-American men ages 45-68 (Honolulu Heart Program)	Unacceptable FEV_1 _measurements at any time point
					
5	Burrows *et al*. 1987 [10], Camilli *et al*. 1987 [21]	Tucson, AZ USA	1972-1973 to 1993	Random sample of 1,705 non-Mexican-American white men and women ages 20+	Asthmatic, FEV_1 _< 60% predicted
					
6	Chambers *et al*. 1999 [22]	Birmingham, England	1987-1996	Inner-city general practice study in 117 men and women ages 45-74	None
					
7	Chinn *et al*. 2005 [23], Sunyer *et al*. 2005 [24]	Western Europe (26 centres), USA (1 centre)	1991-1993 to 2002	Random sample of 6,654 men and women ages 20-44 (European Community Respiratory Health Survey)	None
					
8	Clement and van de Woestijne 1982 [25]	Belgium	1960-1975	2,406 male members of Belgian Air Force ages 20-45	Less than 3 FEV_1 _measurements
					
9	Comstock *et al*. 1970 [26]	USA, four cities	1962-1963 to 1969	527 male telephone workers ages 40-59	Retired or died by follow-up
					
10	Corbin *et al*. 1979 [27]	Montreal, QC, Canada	1971-1976	42 men and women^f ^ages 29-74, smokers attending a smoking cessation clinic, nonsmokers undefined	FEV_1_/FVC <70%
					
11	Eriksson *et al*. 1985 [28]	Malmö, Sweden	1976-1982	Representative sample of 63 men age 50	None
					
12	Ferris *et al*. 1976 [29]	Berlin, NH, USA	1967-1973	1,156 inhabitants of industrial city, mainly ages 25-75^g^	None
					
13	Fletcher *et al*. 1970 [30], Fletcher *et al*. 1976 [6], Fletcher *et al*. 1977 [31]	London, England	1961-1969	792 men in engineering works and clerical workers ages 30-59	Asthmatic, malignant disease, heart disease, tuberculosis, 50% of nonsmokers and 20% of smokers without persistent phlegm or chest illness
					
14	Frew *et al*. (1992) [32]	Vancouver, BC, Canada	1981-1983 to 1989	733 men ages 15+; grain workers, sawmill workers and office workers	Significant medical disorders or FEV_1 _< 1.5 L or severe asthma
					
15	Górecka and Czernicka-Cierpisz 1992 [33]	Warsaw, Poland	1987-1991	116 male and female hospital workers ages 19-71^h^	No direct contact with patients
					
16	Griffith *et al*. 2001 [34]	USA, 4 communities	1989-1993 to 1996	5242 men and women ages 65+ (Cardiovascular Health Study of older adults)	Could not give informed consent, terminally ill, institutionalized, unable to walk, likely to move in next 3 years
					
17	Huhti and Ikkala 1980 [35]	Harjavalta, Finland	1961-1971	1,037 men and women in rural population ages 40-64	Pulmonary tuberculosis, clinically significant respiratory disease (other than asthma or emphysema)
					
18	Humerfelt *et al*. 1993 [36]	Bergen, Norway	1965-1970 to 1990	Random sample of 951 men ages 22-54	None
					
19	Katoh *et al*. 2001 [37]	K-town, Japan	1985-1988 to 2000	1,596 men and women ages 39+	Ever had asthma
					
20	Kauffman *et al*. 1979 [38], Kauffman *et al*. 1979 [39], Kauffman *et al*. 1997 [40]	Paris area, France	1960-1961 to 1972	575 working men ages 30-54	Incorrect spirographs at either survey
					
21	Krzyzanowski *et al*. 1986 [41]	Cracow, Poland	1968-1981	Random sample of 1,824 male and female residents ages 19-70	None
					
22	Lange *et al*. 1989 [42], Vestbo and Lange 1994 [43]	Copenhagen, Denmark	1976-1978 to 1983	Random sample of 7,764 men and women ages 20+ from area around hospital (Copenhagen City Heart Study)	Asthmatic, smoked tobacco products other than cigarettes, quit <1 year before end
					
23	Taylor *et al*. 1985 [44], Lim *et al*. 1988 [45], Watson *et al*. 1993 [46], Watson *et al*. 2000 [47]	West London, England	1974-1997	227 men ages 20-54 recruited from local employers, supplemented by sample of heavy smokers used in earlier studies	Asthmatic, chest illness, abnormal X-ray
					
24	Liu and Wang 1999 [48]	Beijing, China	1987-1997	63 men and women^f ^ages 60+	Abnormal physical examination, ECG or X-ray; FVC ≤80% of pretest; FEV_1 _≤75% of pretest; FEV_1_/FVC ≤75%
					
25	Olofsson *et al*. 1986 [49]	Gothenburg, Sweden	1973-1980	460 men born in 1913 (age 60) or in 1923 (age 50)	None
					
26	Omori *et al*. 2005 [50]	Kumamoto, Japan	1994-1999	1,888 men ages 35-74 attending medical checkup	Asthmatic, other pulmonary disease, X-ray abnormalities, quit smoking before baseline
					
27	Sandvik *et al*. 1995 [51]	Oslo, Norway	1972-1975 to 1982	1,393 healthy men ages 40-59 working in 5 companies	Heart disease, stroke, cancer, diabetes, hypertension and other miscellaneous diseases
					
28	Sato *et al*. 1997 [52]	Niita City, Japan	1983-1986 to 1989	429 "healthy" male office workers ages 15-65	Heart or lung disease
					
29	Sherman *et al*. 1992 [53], Xu *et al*. 1992 [54]	USA, 6 cities	1974-1976 to 1988	Random sample of 8,191 men and women ages 25-74 (Six Cities Study)	None
					
30	Soejima *et al*. 2000 [55]	Tokyo, Japan	1991-1994 to 1999	83 men and women ages 35-83 attending Keio University Hospital	Lung cancer, marked lung abnormalities
					
31	Tashkin *et al*. 1984 [56], Tashkin *et al*. 1994 [57]	Los Angeles, CA, USA	1973-1978 to 1982	2,401 men and women ages 25-64 in 4 areas varying by pollution level	Nonwhites, inconsistent demographics
					
32	Van der Lende *et al*. 1981 [58], Xu *et al*. 1994 [4], Rijcken *et al*. 1995 [5]	Vlagtwedde and Vlaardingen, Netherlands	1965-1969 to 1990	4,692 men and women ages 15-54 in a rural area and a polluted area	None
					
33	Villar *et al*. 1995 [59]	Southampton, England	1987-1988 to 1992	198 men and women ages 65+ randomly selected from 3 general practices	None
					
34	Vollmer *et al*. 1985 [60]	Portland, OR, USA	1974-1983	Random sample of 48 men and women ages 25-54 from Multnomah County	None
					
35	Vollmer *et al*. 1985 [60]	Portland, OR, USA	1971-1972 to 1982	128 men and women ages 34-83^h ^volunteering for lung function testing	Abnormal FEV_1_
					
36	Wilhelmsen *et al*. 1969 [61]	Gothenburg, Sweden	1963-1967	313 men born in 1913 (age 50)	None
					
47	Kohansal *et al*. 2009 [62]	Framingham, MA, USA	1971-1975 to 1977	4,391 men and women ages 13-71 (Framingham Offspring Cohort)	None
					
	Studies of patients with specific diseases				
					
37	Annesi *et al*. 1992 [63]	Paris, France	1980-1981 to 1986	310 male policemen ages 22-55 with some potential risk factors for FEV_1 _decline^i^	None
					
38	Demedts 1988 [64]	Leuven, Belgium	1975-1985	13 male patients ages 41-63 with early emphysema^j^	None
					
39	Grol *et al*. 1999 [65]	Groningen, Netherlands	1983-1986 to 1996	95 men and women ages 21-33 identified as having allergic asthma when assessed at ages 5-14	Other specific respiratory diseases, for example, cystic fibrosis or tuberculosis
					
40	Howard 1974 [66]	Sheffield, England	1966-1972	144 men and women ages 42-78^h ^with obstructive airway disease	Ischaemic and rheumatic heart disease, severe physical deformity
					
41	Hughes *et al*. 1982 [67]	London, England	1966-1976 to 1979	56 men ages 39-71^h ^with emphysema and vascular attenuation or destruction	Other severe lung diseases or abnormalities, thoracic surgery, α_1_-antitrypsin deficiency^k^
					
42	Postma *et al*. 1986 [68]	Groningen, Netherlands	1964-1972 to 1985	81 nonallergic men and women ages 30-66^h ^with chronic airflow obstruction and considerable lung function impairment^l^	Other progressive or life-threatening disease, used corticosteroids for >9 months
					
	Intervention studies				
					
43	Anthonisen *et al*. 1994 [7], Scanlon *et al*. 2000 [69], Kanner *et al*. 2001 [70], Anthonisen *et al*. 2002 [71]	USA and Canada, 10 centres	1986-1989 to 2000	5,887 men and women ages 35-60 with mild to moderate COPD^m ^who smoked 10+ cigarettes/day within 30 days of screening (Lung Health Study). The subjects were randomly allocated to 3 groups: SIA = special intervention smoking cessation programme plus ipratropium bromide inhaler, SIP = special intervention smoking cessation programme plus placebo inhaler, UC = usual care group	Serious illness, pregnant, used physician-prescribed bronchodilators, β-adrenergic antagonists or systemic glucocorticoids or admitted 25+ drinks/week
					
44	Soriano *et al*. 2007 [72]	USA and Europe, 7 trials	Dates not given; 12- to 36-month follow-up period	1,901 men and women in placebo groups of pooled data from 7 randomized trials of inhaled corticosteroids versus placebo in patients with moderate to severe COPD	Asthmatic, ex-smokers (in one of the 7 trials)
					
45	Townsend 1987 [73], Townsend *et al*. 1991 [74]	USA, 22 centres	1973-1974 to 1982	4,926 men ages 35-57 free of heart disease but at high risk based on their blood pressure, serum cholesterol level and cigarette smoking (MRFIT study). The subjects were randomly allocated to 2 groups: SI = special intervention on smoking, diet and antihypertensive medication; UC = usual care	Very high blood pressure or cholesterol, used β-blockers, serious life-threatening disease, believed unable to participate, smoked cigars, cigarettes or pipes, FEV_1 _measured for <2 years in latter half of follow-up
					
46	Xie *et al*. 2001 [75]	China, 3 provinces	1992-2000	869 men and women ages 15+ living in rural areas with chronic respiratory symptoms^n ^and FEV_1_/FVC ≥ 70%. The areas were randomly allocated to intervention, involving establishment or an organization towards improving medical services, promoting smoking cessation and supplying targeted pharmaceutical treatment, and control.	None

^a^FEV_1_, forced expiratory volume in 1 second; FVC, forced vital capacity; ECG, electrocardiogram; COPD, chronic obstructive pulmonary disease; MRFIT study, Multiple Risk Factor Intervention Trial. ^b^Only references to publications providing relevant data are shown. On occasion, additional publications were used to obtain further study details. ^c^The range of years for the baseline evaluation is shown followed by the last year at which follow-up occurred. ^d^The numbers of subjects given are generally numbers followed up. The age range is sometimes estimated approximately from the mean, standard error/deviation and sample size. ^e^Subjects in the age range 7-24 are also included in the study, but the results are not used. ^f^It is assumed that subjects of both sexes were included, though this is not stated in the paper. ^g^Some older subjects previously studied in 1961 were also included. ^h^The age range is approximate, estimated from the mean, standard error/deviation and sample size. ^i^The potential risk factors for FEV_1 _decline("beta") include a history of asthma, wheezing, any perceived hyperresponsiveness symptom, eczema, urticaria, bronchopneumonia before age 2, eosinophilia (among nonsmokers) and heterozygous Z mutation of the α_1_-antitrypsin gene (PiMZ) phenotype. ^j^Diagnosed on the basis of a decrease in single-breath diffusing capacity and in elastic lung recoil with hyperinflation and only minor airway obstruction, and with compatible chest X-ray film changes. ^k^Homozygous for Pi type Z α_1_-antitrypsin deficiency or heterozygous for SZ type α_1_-antitrypsin deficiency. ^l^FEV_1_/FVC 40% to 55% and increasing <15% after bronchodilator. ^m^FEV_1_/FVC ≤70% (screen 2) and ≤75% (screen 3), and FEV_1 _percentage predicted 55% to 90% (screen 2) and 50% to 90% (screen 3). ^n^Cough and/or expectoration for 2+ years for 3+ mo/yr.

**Table 2:** Distribution of study characteristics

Characteristic	Level	No. of studies	(%)	Characteristic	Level	No. of studies	(%)
Sex	Both	28	(59.6)	Year at end^a^	1960-69	3	(6.4)
	Males only	19	(40.4)		1970-79	10	(21.3)
					1980-89	15	(31.9)
Lowest age	13-29	23	(48.9)		1990-99	14	(29.8)
	30-39	10	(21.3)		2000+	4	(8.5)
	40-49	8	(17.0)		Not known^b^	1	(2.1)
	50-59	3	(6.4)				
	60+	3	(6.4)	Follow-up (yr)	4-9	23	(48.9)
					10-14	13	(27.7)
Highest age^c^	< 50	3	(6.4)		15-19	3	(6.4)
	50-59	12	(25.5)		20+	7	(14.9)
	60-69	8	(17.0)		Not known^b^	1	(2.1)
	70+	24	(51.0)				
				Study type	Cohort	43	(91.5)
Country	USA	13	(27.7)		Intervention	4	(8.5)
	Canada	2	(4.3)				
	UK	6	(12.8)	Population	General	37	(78.7)
	France	2	(4.3)		Diseased^d^	10	(21.3)
	Belgium	2	(4.3)				
	Netherlands	3	(6.4)	Medical exclusions	Some	25	(53.2)
	Sweden	3	(6.4)		None	22	(46.8)
	Norway	2	(4.3)				
	Denmark	1	(2.1)	Subjects (at start)	13-100	9	(19.1)
	Finland	1	(2.1)		101-500	10	(21.3)
	Poland	2	(4.3)		501-1,000	7	(14.9)
	Japan	4	(8.5)		1,001-5,000	15	(31.9)
	China	2	(4.3)		> 5,000	6	(12.8)
	Australia	1	(2.1)				
	Multicountry	3	(6.4)	Betas adjusted	None	29	(61.7)
					Some	18	(38.3)
Year at start	1960-69	16	(34.0)				
	1970-79	15	(31.9)				
	1980-89	11	(23.4)				
	1990-99	4	(8.5)				
	Not known^b^	1	(2.1)				

^a^Year at end of follow-up. ^b^Study 44 gave no information on timing, but since it was published in 2007 and involved follow-up of at most 3 years, it seems likely to have been conducted recently. ^c^At baseline. ^d^All four intervention studies comprised patients with specified medical conditions.

### Estimates of FEV_1 _decline by smoking habit ("betas")

From the 47 selected studies, 951 beta estimates were derived. A total of 849 are direct estimates for specified smoking groups, 12 are estimates expressed relative to continuing smokers and 90 are estimates expressed relative to never smokers. For the purposes of this publication, attention is restricted to the direct estimates. This is partly because the number of direct estimates is much larger and partly because both the studies providing data relative to continuing smokers and 8 of 10 studies providing data relative to never smokers also provide direct estimates of betas. Also, though the estimates relative to never smokers can be used to estimate differences in betas between continuing smokers and those who gave up smoking (our major interest), it was not possible to estimate SDs because of the nonindependence of the betas for the two smoking groups arising from the common comparison group.

The analyses conducted are either unweighted or inverse variance-weighted. For a beta to be included in the weighted analyses, an estimate of its SE is required. This information is available directly for 173 of the 849 betas and can readily be calculated from SD and *N *for 205 and from the 95% CI for 44. While it was not possible to derive an estimate for 154 betas, an estimate of SE was imputed for 273 betas where only *N *was available using age-specific estimates of SD (52.75 for age <40, 52.11 for age 40-49, 48.97 for age 50-69 and 26.61 for age 70+) derived from those studies which did provide an estimate of SD.

### Selection of betas for the main analyses comparing the four smoking groups

Of the 849 direct estimates, 97 were not considered for further analysis, as information on smoking habits was unavailable at baseline for 4 betas and at the end of follow-up for 93. Of the remaining 752 estimates, 684 relate to the major smoking groups: never smokers, ex-smokers, quitters and continuing smokers, with the remaining 68 relating to rarer or less clearly defined combinations of smoking habits, such as those who were never smokers at baseline and current smokers at follow-up or those who were current smokers at baseline and ever smokers at follow-up.

A further 173 betas were also rejected from the main analysis: 166 because they were dose-response estimates (considered separately) and 7 because the smokers were smokers of pipes and/or cigars only. This left 511 potentially useful estimates.

To avoid double-counting of nonindependent data, there was a need in certain studies for a decision to be made regarding which estimates to include in our main analysis and which to exclude. In studies 41 and 43, preference is given to betas on the basis of measurements taken without use of a bronchodilator, as this applies to most of the available data. Preference is also given to betas based on the longest available follow-up time (relevant to studies 1, 4 and 23), betas given separately by age (studies 4 and 25), betas that are adjusted for the most variables (studies 3, 13 and 46), betas from publications that provide information for all four smoking groups (studies 5, 23, 29, 31 and 45) and betas with information on *N*, SD or SE (study 7). Also, for study 32, preference is given to the unstratified data from the 1994 paper [4] for quitters and to the data stratified by airway responsiveness from the 1995 paper [5] for the other smoking groups, as the later source did not give data for quitters. For study 47, preference is given to the data unstratified for healthy versus unhealthy status, as stratified results were available only for current smokers.

The betas and SEs used in the main analyses are shown in Table 3. These relate to only 39 of the 47 studies, with two studies (8 and 33) providing only estimates relative to never smokers and six studies (11, 14-16, 24 and 40) having incomplete information on smoking habits. For some studies, data are not available for all four smoking groups, notably for study 25, where only data for never smokers are available, data for continuing smokers being classified by amount smoked and for those who gave up being for ex-smokers and quitters combined. Studies 37, 46 and 47 also have data only for ex-smokers and quitters combined. It should be noted that for study 29, the age-specific data which have no SEs are used in the unweighted analyses, but the ages combined data, which do have SEs, are used in the weighted analyses. SEs are available for all other estimates except for those for never smokers and ex-smokers in study 45. There are a total of 303 estimates of beta in Table 3 with 295 being available for unweighted analyses (all except the eight estimates for ages 25-74 for study 29) and 261 being available for weighted analyses (all except the 40 estimates in study 29 and the 2 estimates in study 45 without SEs).

**Table 3:** Data on FEV1 decline selected for main analyses

		Stratifying variables			Follow-up (yr)	No. of adjusted	Beta (SE)^d^			
					
Study **no**.	Reference	Sex	Age range^a^	Other^b^		variables^c^	Never^e ^smokers	Ex-smokers^f^	Quitters^g^	Continuing smokers^h^
1	Bartholomew and Knuiman [12]	M	19-44	-	6	2	36.8 (6.2)		14.0 (11.2)	54.7 (7.1)
		M	45+	-	6	2	45.8 (6.4)		51.2 (7.9)	60.5 (6.5)
		F	19-44	-	6	2	24.3 (6.6)		-15.3 (11.3)	13.7 (10.8)
		F	45+	-	6	2	30.7 (4.7)		35.2 (7.4)	46.5 (10.3)
2	Beck *et al. *[14]	M	25-34	-	6	0	-20.7 (9.5)	0.8 (12.1)		-3.0 (9.8)
		M	35-44	-	6	0	2.5 (15.0)	-14.3 (8.8)		19.5 (9.1)
		M	45+	-	6	0	20.0 (4.9)	31.0 (3.4)		25.5 (5.3)
		F	25-34	-	6	0	-8.5 (6.9)	-32.5 (11.5)		15.2 (8.3)
		F	35-44	-	6	0	18.8 (7.5)	13.5 (12.0)		20.8 (8.6)
		F	45+	-	6	0	10.3 (2.9)	10.7 (5.0)		32.0 (5.8)
3	Bossé *et al. *[16]	M	20-34	-	5	1	23.0 (9.0)		24.0 (11.0)	57.0 (8.0)
		M	35-42	-	5	1	47.0 (7.0)		61.0 (12.0)	76.0 (6.0)
		M	43-80	-	5	1	82.0 (7.0)		73.0 (9.0)	101.0 (7.0)
4	Burchfiel *et al. *[11]	M	45-49	-	6	1	19.5 (2.9)	19.8 (3.1)	21.2 (4.9)	31.3 (2.4)
		M	50-59	-	6	1	21.6 (1.8)	21.5 (2.0)	28.3 (3.3)	32.4 (1.8)
		M	60-68	-	6	1	25.0 (3.2)	26.0 (3.4)	35.1 (6.1)	40.1 (3.5)
5	Camilli *et al. *[21]	M	20-34	-	9.4	0	1.0 (6.1)	-9.0 (15.2)	-22.0 (12.4)	6.0 (7.2)
		M	35-49	-	9.4	0	9.0 (8.3)	1.0 (9.8)	12.0 (13.5)	18.0 (7.6)
		M	50-69	-	9.4	0	19.0 (7.1)	24.0 (5.4)	34.0 (10.0)	40.0 (5.8)
		M	70+	-	9.4	0	25.0 (3.8)	26.0 (3.2)	37.0 (7.7)	26.0 (7.7)
		F	20-34	-	9.4	0	-5.0 (5.5)	-18.0 (13.2)	-27.0 (14.1)	-4.0 (8.0)
		F	35-49	-	9.4	0	6.0 (6.5)	4.0 (9.1)	7.0 (17.4)	12.0 (7.4)
		F	50-69	-	9.4	0	13.0 (3.9)	15.0 (5.8)	17.0 (9.1)	21.0 (5.0)
		F	70+	-	9.4	0	21.0 (2.1)	20.0 (4.6)	25.0 (7.7)	26.0 (6.1)
6	Chambers *et al. *[22]	M+F	45-74	-	9	0	43.5 (4.4)	47.3 (6.4)	41.1 (19.3)	52.0 (4.4)
7	Chinn *et al. *[23]	M	20-44	-	8	0	32.0 (1.1)	31.0 (1.6)	31.0 (2.1)	35.0 (1.4)
		F	20-44	-	8	0	24.0 (0.7)	27.0 (1.2)	22.0 (1.8)	27.0 (1.1)
9	Comstock *et al. *[26]	M	40-59	-	5.25	0			32.4 (7.1)	78.1 (3.1)
10	Corbin *et al. *[27]	M+F	29-74^i^	-	4^j^	0	-8.6 (15.0)		35.0 (16.3)	20.0 (11.4)
12	Ferris *et al. *[29]	M	25-74	-	6	2	-10.0 (4.9)	-6.7 (4.5)		
		F	25-74	-	6	2	-8.3 (2.6)	0.0 (7.2)		
13	Fletcher *et al. *[6]	M	30-59	F1	8	4	36.0 (9.0)	34.0 (6.0)	37.0 (11.0)	60.5 (2.2)
		M	30-59	F2	8	4	45.0 (6.0)	36.0 (5.0)	47.0 (11.0)	47.6 (2.3)
		M	30-59	F3	8	4	33.0 (3.0)	26.0 (4.0)	26.0 (14.0)	40.4 (2.2)
17	Huhti *et al. *[76]	M	40-64	-	10	0	33.0 (3.4)	45.0 (3.1)	44.0 (4.0)	51.0 (2.4)
		F	40-64	-	10	0	27.0 (1.0)	27.0 (4.7)	39.0 (6.9)	35.0 (3.5)
18	Humerfelt *et al. *[36]	M	22-54	-	23	0	46.6 (1.4)	47.0 (1.3)	51.5 (1.1)	
19	Katoh *et al. *[37]	M	39+	-	12	0	30.0 (1.9)	31.7 (1.8)	37.9 (2.1)	39.2 (1.5)
		F	39+	-	12	0	20.8 (0.5)	21.7 (2.5)	16.7 (5.2)	30.8 (4.2)
20	Kauffmann *et al. *[39,40]	M	30-54	-	12	2^k^		40.8 (4.8)	49.4 (4.1)	46.1 (1.6)
21	Krzyzanowski *et al. *[41]	M	19-70	-	13	2	47.3 (4.1)	50.4 (5.3)	66.5 (4.4)	59.7 (2.6)
		F	19-70	-	13	2	38.3 (1.9)	30.1 (9.6)	37.3 (6.9)	42.0 (3.6)
22	Lange *et al. *[42]	M	20-54	-	5	0	21.0 (7.0)	27.0 (7.0)		
		M	55+	-	5	0	34.0 (9.0)	36.0 (5.0)		
		F	20-54	-	5	0	13.0 (3.0)	18.0 (5.0)		
		F	55+	-	5	0	32.0 (3.0)	32.0 (4.0)		
23	Watson *et al. *[47]	M	20-54	-	22	0	34.2 (2.2)	33.1 (2.8)	38.8 (3.3)	51.0 (4.0)
25	Olofsson *et al. *[49]	M	50	-	7	0	54.3 (8.5)			
		M	60	-	7	0	57.1 (6.3)			
26	Omori *et al. *[50]	M	35-44	-	5	0	32.8 (8.3)		38.8 (6.1)	33.7 (4.0)
		M	45-54	-	5	0	31.9 (2.9)		35.3 (2.7)	44.5 (2.1)
		M	55-64	-	5	0	31.3 (2.9)		35.4 (2.7)	38.9 (2.7)
		M	65-74	-	5	0	33.9 (3.1)		30.4 (4.2)	43.1 (4.5)
27	Sandvik *et al. *[51]	M	40-49	-	7^l^	0			10.3 (6.4)	31.7 (4.3)
		M	50-59	-	7^l^	0			27.4 (4.8)	45.4 (4.8)
28	Sato *et al. *[52]	M	15-65^m^	-	3	0	2.0 (12.4)	18.0 (16.9)		36.0 (7.9)
29	Xu *et al. *[54]	M	25-34	-	6	1	15.2	6.4	-6.2	34.2
		M	35-44	-	6	1	24.9	23.9	47.4	34.5
		M	45-54	-	6	1	41.9	39.4	32.2	63.8
		M	55-64	-	6	1	45.9	43.2	66.7	60.4
		M	65-78	-	6	1	55.6	52.3	60.2	63.3
		M	25-74	-	6	2	37.8 (2.0)	34.3 (1.8)	41.2 (5.0)	52.9 (2.0)
		F	25-34	-	6	1	13.8	13.9	-7.4	22.2
		F	35-44	-	6	1	25.0	29.8	6.2	34.9
		F	45-54	-	6	1	30.4	26.0	31.7	40.1
		F	55-64	-	6	1	34.1	40.6	38.9	48.3
		F	65-78	-	6	1	39.2	34.9	70.1	38.2
		F	25-74	-	6	2	29.0 (0.9)	29.6 (1.6)	28.7 (4.3)	38.0 (1.2)
30	Soejima *et al. *[55]	M+F	35-83^n^	-	5	0	20.0 (1.7)	30.0 (8.7)		60.0 (3.4)
31	Tashkin *et al. *[56]	M	25-64	-	5	3	56.0 (2.6)	52.0 (3.0)	62.0 (5.1)	70.0 (3.1)
		F	25-64	-	5	3	42.0 (1.9)	38.0 (2.8)	38.0 (6.6)	54.0 (4.0)
32	Xu *et al. *[4]	M	25-54	-	24	0			6.1 (7.2)	
		F	25-54	-	24	0			2.7 (6.1)	
	Rijcken *et al. *[5]	M	25-54	H0	24	1	30.8 (9.8)	28.5 (5.0)		37.1 (4.5)
		F	25-54	H0	24	1	24.9 (3.9)	24.7 (7.2)		27.3 (5.1)
		M	25-54	H1	24	1	41.2 (17.0)	33.0 (8.0)		40.3 (6.5)
		F	25-54	H1	24	1	25.3 (6.0)	30.6 (9.6)		33.1 (6.4)
34	Vollmer *et al. *[60]	M+F	25-54	B1	9	0	38.0 (52.1)		89.0 (19.7)	67.0 (26.1)
		M+F	25-54	B0	9	0	43.0 (30.1)		43.0 (11.4)	45.0 (15.0)
35	Vollmer *et al. *[60]	M+F	34-83	B1	11	0	49.0 (18.5)		70.0 (17.3)	70.0 (11.9)
		M+F	34-83	B0	11	0	37.0 (10.7)		56.0 (10.0)	60.0 (6.9)
36	Wilhelmsen *et al. *[61]	M	50	-	4	0			40.0 (16.8)	73.2 (7.1)
37	Annesi *et al. *[63]	M	22-55	I1	5	0	31.7 (5.9)			51.7 (5.8)
		M	22-55	I2	5	0	59.7 (9.8)			32.7 (8.8)
38	Demedts [64]	M	41-63^o^	-	10	0		72.5 (37.5)	88.8 (21.4)	98.4 (10.8)
39	Grol *et al. *[65]	M+F	21-33	-	11	0	19.7 (8.0)		-26.4 (23.6)	23.4 (11.5)
41	Hughes *et al. *[67]	M	44-69	-	8	0		16.4 (8.8)		
42	Postma *et al. *[68]	M+F	30-66	-	11	0			49.0 (7.0)	85.0 (5.1)
43	Kanner *et al. *[70]^p^	M+F	35-60	D1	5	0		13.1 (1.8)		55.9 (1.0)
		M+F	35-60	D2	5	0		27.6 (5.7)		58.5 (3.0)
		M+F	35-60	D3	5	0		24.0 (7.8)		63.4 (3.0)
		M+F	35-60	D4	5	0		20.3 (8.7)		66.4 (3.7)
		M+F	35-60	D5	5	0		12.0 (11.9)		69.4 (4.7)
44	Soriano *et al. *[72]^q^	M	40+	-	1.5	1		16.0 (2.7)		21.3 (2.0)
		F	40+	-	1.5	1		11.3 (4.3)		16.7 (2.0)
45	Townsend [73]^p^	M	35-57	-	7	0	39.5	40.0	40.0 (4.7)	62.3 (2.8)
46	Xie *et al. *[75]^p^	M+F	15+	-	8	5	39.5 (3.3)			37.3 (2.2)
47	Kohansal *et al. *[62]	M	13-71	-	23	0	19.6 (1.3)			38.2 (2.2)
		F	13-71	-	23	0	17.6 (1.9)			23.9 (1.6)

^a^Except where noted, age range applies to results for each smoking group. ^b^Other stratifying variables are indicated as follows: F1, F2, F3 = forced expiratory volume in 1 second (FEV_1_) at baseline <55, 55-64, 65+ cL/m^3 ^H0, H1 = histamine responsiveness (no or yes); B0, B1 = bronchodilator responsiveness (no or yes); I1, I2 = immunoglobulin E level ≤100, 100+ IU/mL; D1, D2, D3, D4, D5 = doctor visits for lower respiratory illnesses 0-0.24, 0.25-0.49, 0.50-0.99, 1.00-1.49, or 1.50+ per year. ^c^Number of adjustment variables. The variables considered by study are 1: age, change in body mass index; 3: baseline FEV_1_; 4: height; 12: height; 13: age, height, season, and observer; 29: age (only for age group 25-74), height; 31: age, height, area; 32: height; 46: sex, age, height, region, family history of chronic obstructive pulmonary disease (COPD). ^d^Beta = Decline in FEV_1 _(in mL/yr) over follow-up period. Results are not postbronchodilator measurements, except for study 44. For studies 9, 22 and 38, smoking is of cigarettes only. For studies 2, 3, 12, 23, 26, 29, 31 and 43, smoking is of cigarettes regardless of other products. For study 13, current smoking is of cigarettes regardless of other products, but giving up smoking is of any product. For other studies, smoking relates to any tobacco product. ^e^Never smoked by end of follow-up. ^f^Gave up smoking before baseline and did not resume smoking. ^g^Smoked at baseline, but not at end of follow-up. ^h^Smoked at baseline and at end of follow-up. ^i^Age range varies by smoking group: Never smokers 30-65, quitters 30-74 and continuing smokers 29-60. ^j^Follow-up period is 3.5 years for never smokers. ^k^Only results for quitters and continuing smokers were adjusted. ^l^Follow-up period is 7.6 years for quitters and 7.1 years for continuing smokers. ^m^Age range varies by smoking group: Never smokers 15-60, ex-smokers 26-64 and continuing smokers 21-61. ^n^Age range varies by smoking group: Never smokers 32-80, ex-smokers 29-91, continuing smokers 38-78. ^o^Age range varies by smoking group: Never smokers 44-51, ex-smokers 42-63 and continuing smokers 41-53. ^p^Results are based on all groups combined in the intervention study. ^q^Results are based only on usual care group and are postbronchodilator measurements.

### Comparison of betas in the four smoking groups

Table 4 compares betas in the four smoking groups based on unweighted and inverse variance-weighted analysis. The results are shown without adjustment, with adjustment for age and sex and with adjustment for block. Each row of data in Table 3 is a block, and the block-adjusted analysis attempts to adjust simultaneously for all the factors fixed in the study design and by the choice of subgroup for analysis. Adjustment for age and sex reduces the residual variance by 27.4% in the unweighted analysis and by 30.6% in the weighted analysis, while adjustment for block reduced it by 76.9% in the unweighted analysis and by 78.1% in the weighted analysis.

**Table 4:** FEV1 decline (in mL/yr) by smoking group^a^

		Never smokers^b^ (95% CI)	Ex-smokers^c^ (95% CI)	Quitters^d^ (95% CI)	Continuing smokers^e^ (95% CI)
Unweighted analysis^f^					
Number of betas		80	66	64	85
					
Unadjusted	Mean	27.9 (23.5-32.4)	24.1 (19.2-29.1)	33.4 (28.4-38.4)	42.8 (38.5-47.2)
	Diff 1	-14.9***	-18.7***	-9.4**	Base
	Diff 2	Base	-3.8^NS^	+5.5^NS^	+14.9***
					
Adjusted for age and sex	Mean	28.8 (25.0-32.7)	24.6(20.3-28.8)	33.0 (28.7-37.3)	41.9 (38.2-45.6)
	Diff 1	-13.1***	-17.4***	-8.9**	Base
	Diff 2	Base	-4.3^NS^	+4.2^NS^	+13.1***
					
Adjusted for block^g^					
(fixed-effects model)	Mean	28.7 (26.4-30.9)	27.0 (24.5-29.5)	30.4 (27.9-33.0)	42.1 (40.0-44.3)
	Diff 1	-13.5***	-15.2***	-11.7***	Base
	Diff 2	Base	-1.7^NS^	+1.7^NS^	+13.5***
					
(random-effects model)	Mean	28.6 (24.4-32.9)	26.6 (22.2-31.0)	30.6 (26.2-35.1)	42.2 (38.0-46.4)
	Diff 1	-13.6***	-15.6***	-11.6***	Base
	Diff 2	Base	-2.1^NS^	+2.0^NS^	+13.6***
					
Weighted analysis^h^					
Number of betas		71	57	56	77
					
Unadjusted	Mean	26.1 (23.9-28.3)	29.3 (25.6-32.9)	38.3 (33.5-43.2)	41.8 (39.1-44.6)
	Diff 1	-15.7***	-12.6***	-3.5^NS^	Base
	Diff 2	Base	+3.1^NS^	+12.2***	+15.7***
					
Adjusted for age and sex	Mean	28.9 (27.0-30.9)	27.9 (24.8-30.9)	34.2 (30.1-38.4)	39.5 (37.2-41.9)
	Diff 1	-10.6***	-11.6***	-5.3*	Base
	Diff 2	Base	-1.1^NS^	+5.3*	+10.6***
					
Adjusted for block^g^					
(fixed-effects model)	Mean	29.2 (28.1-30.4)	27.6 (25.9-29.4)	31.6 (29.1-34.1)	40.1 (38.6-41.5)
	Diff 1	-10.8***	-12.4***	-8.5***	Base
	Diff 2	Base	-1.6^NS^	+2.4^NS^	+10.8***
					
(random-effects model)	Mean	29.6 (25.6-33.5)	28.0 (23.9-32.1)	32.2 (27.8-36.7)	40.8 (36.9-44.8)
	Diff 1	-11.3***	-12.8***	-8.6***	Base
	Diff 2	Base	-1.5^NS^	+2.7^NS^	+11.3***

^a^Based on data in Table 3. The table shows the mean forced expiratory volume in 1 second (FEV_1_) decline in millilitres per year in the four smoking groups with 95% confidence interval (95% CI) and also the mean differences from continuing smokers (Diff 1) and from never smokers (Diff 2), with statistical significance indicated. ***P < 0.001, **P < 0.01, *P < 0.05 and NS *P *≥ 0.05. ^b^Never smoked by end of follow-up. ^c^Gave up smoking before baseline and did not resume smoking. ^d^Smoked at baseline, but not at end of follow-up. ^e^Smoked at baseline and at end of follow-up. ^f^Using all estimates in Table 3 except the 8 estimates for ages 25-74 for study 29. ^g^A block is a row in Table 3 consisting of a set of results for the same study and stratifying variables. ^h^Using all estimates in Table 3, except the 40 estimates in study 29 and the 2 estimates in study 45 without SEs.

The results consistently show a lower beta in never smokers than in continuing smokers (*P *< 0.001) with the difference estimated as 10.8 mL/yr (95% CI, 8.9 to 12.8) in the weighted, block-adjusted analysis and over 10 mL/yr in all the other analyses. Ex-smokers also show a beta that is consistently less than that in continuing smokers (*P *< 0.001 in all analyses), with the estimated difference (12.4 mL/yr; 95% CI, 10.1 to 14.7, in the weighted, block-adjusted analysis) again always over 10 mL/yr. Betas for ex-smokers and never smokers do not vary significantly in any of the analyses. Betas for quitters lie between those for continuing smokers and never smokers in all the analyses, consistent with the quitters' having smoked for only part of the follow-up period. In the weighted, block-adjusted analysis, beta is estimated to be 8.5 mL/yr (95% CI, 5.6 to 11.4) less in quitters than in continuing smokers (*P *< 0.001) and 2.4 mL/yr (95% CI, -0.4 to 5.1) more in quitters than in never smokers.

In the text above, the block-adjusted results cited are those based on fixed-effects modelling. As is evident from Table 4 the alternative analyses using random-effects modelling produced virtually identical beta estimates to those using fixed-effects modelling. Though the CIs using random-effects modelling are somewhat wider, the interpretation of the data is unaffected, with betas for continuing smokers clearly greater (*P *< 0.001) than those in the other three groups, which do not differ significantly.

Similar conclusions were also reached using block-adjusted analyses (fixed effects or random effects) which were run excluding those betas where only beta and *N *were available, and the SE was imputed from age-specific estimates of SD derived from other studies (data not shown).

Two sets of additional analyses corresponding to those in Table 4 but differing in the betas included, were also conducted. The additional weighted analyses were based on those 253 betas used in the unweighted analyses presented in Table 4 that had SEs, and the additional unweighted analyses were based on the 261 betas used in the weighted analyses in Table 4. The results (not shown) were very similar to those shown in Table 4 with mean betas lowest (and similar) in never smokers and ex-smokers, highest in continuing smokers and intermediate in quitters.

### Heterogeneity of betas

The ratio of between-study to within-study variance in betas was estimated as 11.94 for never smokers (*P *< 0.001), 15.51 for continuing smokers (*P *< 0.001), 9.84 for quitters (*P *< 0.01) and 1.32 (NS) for ex-smokers. These ratios were based on, respectively, those 28, 30, 27 and 24 beta estimates considered in the main analyses for which information was available on *N *and also either SE or SD.

### Relationship of beta to age, sex and study characteristics

Table 5 gives the results of analyses relating beta to age, sex and various study characteristics. Note that as some betas were for the sexes combined, the parameter of sex has three levels (males, females, and both). In the inverse variance-weighted analysis, betas are clearly lower in females (compared to both males and the sexes combined) and are lower at age <40 years and age 70+ years than at ages 40-49 and 50-59 years. After adjustment for age, sex and smoking group, no significant association is seen with population type or study type. There is evidence that betas are somewhat greater in studies in Europe than in North America and in studies where the final follow-up year and publication year were 1980 or later. In the unweighted analyses, there are again associations with age, sex, continental location of study and final follow-up year, but the association with publication year is no longer significant.

**Table 5:** Relationship of beta (in mL/yr) to study characteristics adjusted for smoking group, sex and age

		Unweighted analysis			Inverse variance-weighted analysis		
Factor	Level	Number of betas	Beta (95% CI)	*P *value^a^	Number of betas	Beta (95% CI)	*P *value^a^
Sex^b^	Males	168	35.8 (33.1-38.4)	Base	150	34.6 (32.7-36.5)	Base
	Females	88	23.9 (20.2-27.7)	---	72	28.3 (26.3-30.3)	---
	Both	39	38.2 (32.5-43.9)	NS	39	40.7 (36.2-45.3)	+
							
Age^c^	< 40	47	13.2 (8.2-18.3)	Base	39	28.7 (25.8-31.5)	Base
	40-49	78	33.9 (30.0-37.8)	+++	68	42.8 (39.6-46.1)	+++
	50-69	123	37.8 (34.7-41.0)	+++	115	32.1 (30.1-34.1)	NS
	70+	47	35.8 (30.7-40.9)	+++	39	27.9 (25.2-30.6)	NS
							
Length of follow-up period	Per year	295	-0.16 (-0.57 to 0.25)	NS	261	-0.03 (-0.27 to 0.21)	NS
							
Continent	North America	148	30.3 (27.5-33.1)	Base	114	29.0 (26.1-32.0)	Base
	Europe	85	36.7 (32.9-40.5)	++	85	34.8 (31.5-38.1)	++
	Asia	28	26.3 (19.7-32.8)	NS	28	31.6 (28.2-35.1)	NS
	Australasia	12	41.9 (31.6-52.1)	+	12	42.1 (27.5-56.7)	NS
	Multicountry	22	34.9 (27.2-42.7)	NS	22	34.3 (30.0-38.6)	NS
							
Final follow-up year	< 1980	78	27.0 (23.3-30.6)	Base	78	25.7 (22.8-28.6)	Base
	1980-1989	98	42.3 (39.1-45.4)	+++	64	37.9 (34.8-41.1)	+++
	1990-1999	91	26.0 (22.7-29.3)	NS	91	27.6 (25.0-30.3)	NS
	2000+	28	35.6 (29.3-42.0)	+	28	35.8 (33.1-38.4)	+++
							
Publication year	< 1980	26	27.1 (20.1-34.1)	Base	26	26.3 (20.5-32.0)	Base
	1980-1989	129	33.2 (30.2-36.3)	NS	129	35.0 (32.1-37.9)	++
	1990-1999	99	34.2 (30.7-37.6)	NS	65	35.1 (32.5-37.8)	++
	2000+	41	30.0 (24.4-35.5)	NS	41	30.2 (27.9-32.4)	NS
							
Population type	General	266	32.5 (30.4-34.6)	Base	232	32.7 (31.4-34.1)	Base
	Diseased	29	32.9 (25.8-40.1)	NS	29	28.3 (22.3-34.4)	NS
							
Study type	Prospective	275	32.9 (30.8-35.0)	Base	243	32.7 (31.4-34.1)	Base
	intervention	20	27.6 (19.2-36.0)	NS	18	28.4 (22.6-34.3)	NS

^a^+++, ---*P *< 0.001; ++, -- *P *< 0.01; +, - *P *< 0.05; NS *P *≥ 0.05 with positive signs indicating significantly higher betas than the base level and negative signs indicating significantly lower betas. ^b^Adjusted for smoking group and age only. ^c^Adjusted for smoking group and sex only.

Although Table 5 provides evidence of variation in beta by sex, age and other study characteristics, it was clear from inspection of the SDs in the various regression models that none of these characteristics could explain more than a small part of the between-study heterogeneity noted above (data not shown).

### Differences in betas between continuing smokers and those who gave up smoking

The data in Table 3 allow the calculation of 63 within-block differences in beta between continuing smokers and quitters and 60 within-block differences between continuing smokers and ex-smokers. Of the 123 differences, 21 have no SE (20 in study 29 and 1 in study 45), so these data could not be used in weighted analyses. The mean difference between continuing smokers and quitters is estimated as 11.2 mL/yr (95% CI, 7.0 to 15.3), based on unweighted analysis of 61 betas, and 7.1 mL/yr (95% CI, 4.7 to 9.6) based on weighted analysis of 53 betas, while the difference between continuing smokers and ex-smokers is estimated as 14.9 mL/yr (95% CI, 11.5 to 18.3) based on unweighted analysis of 58 betas and as 12.3 mL/yr (95% CI, 8.7 to 16.0) based on weighted analyses of 49 betas.

### Variation by age, sex and study characteristics in differences in beta between continuing smokers and those who gave up smoking

There is little consistent evidence that differences in beta between continuing smokers and those who gave up smoking vary meaningfully by age (adjusted for sex) or sex (adjusted for age). There is significant variation by sex (*P *< 0.001) in the differences in betas between continuing smokers and ex-smokers in both the unweighted and inverse variance-weighted analyses, but these differences are due to larger differences where estimates are for the sexes combined, with five of the seven estimates deriving from one study (43). No significant variation is seen by sex in the difference in beta between continuing smokers and quitters. Variation by age (*P *< 0.001) in the difference in beta between continuing smokers and ex-smokers is seen in the weighted analyses, but this does not follow any trend, with the differences being larger for ages 40-49 (18.7 mL/yr, based on *n *= 12 betas) and ages 50-69 (14.5 mL/yr, *n *= 23) than for ages <40 (6.3 mL/yr, *n *= 6) and ages 70+ (9.9 mL/yr, *n *= 8), and is not evident in the unweighted analyses. Variation by age is also evident for the difference between continuing smokers and quitters, but this is evident only in the unweighted analyses (*P *< 0.001), with differences being larger for ages <40 (28.2 mL/yr, *n *= 10) than for ages 40-49 (7.8 mL/yr, *n *= 17), ages 50-69 (10.2 mL/yr, *n *= 25) or ages 70+ (1.1 mL/yr, *n *= 9).

After adjustment for age and sex, there is little evidence of variation in either difference by length of follow-up period, continental location of study, final follow-up year, year of publication or study type. There is, however, a consistent tendency for the difference to be greater where the estimates relate to patients with specific respiratory diseases than where they relate to the general population. For the difference between continuing smokers and quitters, the excess difference associated with having respiratory disease is 21.6 mL/yr (*P *< 0.05) in unweighted analyses (using 61 betas, with 3 relating to subjects with respiratory disease) and 31.3 mL/yr (*P *< 0.05) in inverse variance-weighted analyses (using 53 betas, 3 for subjects with respiratory disease). For the difference between continuing smokers and ex-smokers, the excess is 12.3 mL/yr (*P *< 0.05) in unweighted analyses (using 58 betas, 8 for subjects with respiratory disease) and 4.8 mL/yr (not significant) in weighted analyses (using 49 betas, 8 for subjects with respiratory disease).

### Relationship of beta to amount smoked in continuing smokers

As noted above, 166 of the estimates of beta relate to dose-response relationships. Table 6 presents the data for those 74 estimates which concern continuing smokers, where the unit of exposure is cigarettes per day and where the subjects stayed in the same exposure group between the start and end of follow-up. Eighteen estimates were excluded because they concerned quitters, and 44 were excluded because the unit of exposure was not cigarettes per day or the level of exposure was undefined at follow-up. Of the remaining 104 estimates, 30 were excluded and of those, 12 were excluded because the level of exposure differed between start and follow-up and 18 were excluded (in studies 13 and 31) as preference was given to estimates adjusted for the most variables. Of the 74 betas shown, 36 are from study 29, with 30 individual age betas without CI and 6 combined age estimates with CI. CIs are available for all the other 38 estimates derived from 6 studies. There are 26 "blocks" of independent dose-response relationships.

**Table 6:** Data on FEV1 decline (in mL/year) in continuing smokers by amount smoked^a^

		Stratifying variables				
**Study no**.	Reference	Sex	Age	Other^b^	Amount smoked **(cigarettes/day)**^**c**^	Beta (SE)
4	Burchfiel *et al. *[11]	M	45-68	-	1-19, 20-29, 30-39, 40+	29.8 (3.5), 32.4 (2.0), 35.5 (2.8), 34.3 (2.7)
						
12	Ferris *et al. *[29]	M	25-74	-	1-24, 25+	0.0 (5.7), 6.7 (5.8)
		F	25-74	-	1-24, 25+	1.7 (4.4), 1.7 (8.3)
						
13	Fletcher *et al. *[6]	M	30-59	F1	1-5, 6-15, 16-25, 26+	45.0 (5.0), 55.0 (4.0), 74.0 (5.0), 63.0 (6.0)
		M	30-59	F2	1-5, 6-15, 16-25, 26+	37.0 (6.0), 49.0 (4.0), 51.0 (4.0), 45.0 (10.0)
		M	30-59	F3	1-5, 6-15, 16-25, 26+	39.0 (4.0), 39.0 (4.0), 38.0 (5.0), 52.0 (11.0)
						
22	Lange *et al. *[42]	M	20-54	-	1-14, 15+	22.0 (6.0), 42.0 (5.0)
		M	55+	-	1-14, 15+	52.0 (3.0), 56.0 (6.0)
		F	20-54	-	1-14, 15+	17.0 (4.0), 30.0 (4.0)
		F	55+		1-14, 15+	39.0 (3.0), 48.0 (5.0)
						
29	Xu *et al. *[54]	M	25-34	-	1-14, 15-24, 25+	36.5, 20.6, 41.7
		M	35-44	-	1-14, 15-24, 25+	30.4, 40.3, 39.0
		M	45-54	-	1-14, 15-24, 25+	55.0, 57.4, 68.8
		M	55-64	-	1-14, 15-24, 25+	49.0, 53.8, 68.5
		M	65-78	-	1-14, 15-24, 25+	66.8, 54.1, 73.4
		M	25-74	-	1-14, 15-24, 25+	37.4 (6.0), 47.2 (3.6), 59.9 (3.5)
		F	25-34	-	1-14, 15-24, 25+	16.5, 21.9, 28.0
		F	35-44	-	1-14, 15-24, 25+	28.9, 35.8, 36.9
		F	45-54	-	1-14, 15-24, 25+	32.5, 45.7, 37.8
		F	55-64	-	1-14, 15-24, 25+	42.7, 52.0, 50.4
		F	65-78	-	1-14, 15-24, 25+	29.4, 47.9, 37.4
		F	25-74	-	1-14, 15-24, 25+	31.2 (2.4), 42.0 (1.8), 38.9 (2.5)
						
31	Tashkin *et al. *[57]	M	25-59	-	1-20, 21+	77.8 (6.2), 81.8 (5.0)
		F	25-59	-	1-20, 21+	52.3 (3.3), 61.7 (3.7)
						
32	Xu *et al. *[4]	M	25-54	-	1-14, 15-24, 25+	18.8 (5.3), 26.3 (4.2), 33.2 (4.9)
		F	25-54	-	1-14, 15-24, 25+	15.0 (4.0), 20.4 (5.1), 30.1 (7.6)

^a^For details of length of follow-up and number of adjustment variables, see Table 3. ^b^Other stratifying variables are indicated as follows: F1, F2, F3 = forced expiratory volume in 1 second (FEV_1_) at baseline <55, 55-64, 65+ cL/m^3^. ^c^For the purposes of estimating mean cigarettes/day for an interval, a simple average of the upper and lower limits was used, with the upper limit for the final interval taken as 50 cigarettes/day.

Some dose relationships are evident from inspection, with the betas being higher for the highest consumption than for the lowest consumption in every block, except for one block where the betas are the same. After adjustment for block, the estimated increase in beta per cigarette per day is 0.33 (95% CI, 0.22 to 0.44) in unweighted analysis based on 68 betas and 0.33 (95% CI, 0.20 to 0.45) in inverse variance-weighted analysis based on 44 betas.

### Relationship of some other factors to beta and differences in betas between smoking groups

A number of the studies present betas separately by level of factors other than age, sex or study characteristics. Table 7 summarizes trends in beta by level for those factors for which data were available for continuing smokers and also for quitters and/or ex-smokers. With the notable exception of study 4, which reports a strong tendency for beta to decline with reducing FEV_1_, both in continuing smokers and quitters, the results from studies 5, 13 and 45 are consistent in demonstrating a strong tendency for betas in all three smoking groups to be greater where there is evidence of reduced lung function as determined by low FEV_1_, forced vital capacity (FVC) or presence of mild obstruction. The trend in betas is generally greater in continuing smokers than in quitters or ex-smokers, though the difference in trends between continuing smokers and either quitters or ex-smokers is significant in only one case: study 5, in which a marked trend is seen in continuing smokers, but no trend is seen in ex-smokers.

**Table 7:** Trends in betas (mL/yr) by level of various factors^a^

							Trend (SE)^b^		
							
Factor	Levels	**Study no**.	**Ref**.	Sex	Age range at baseline		Continuing smokers	Quitters	Ex-smokers
Baseline FEV_1 _(cL/m^3^)	65+, 55-64, 1-54	13	Fletcher *et al. *[6]	Male	30-59	Mean	10.0 (1.5)***	4.0 (8.8)	4.8 (3.5)
						Diff^c^	Base	6.2 (8.9)	5.1 (3.8)
									
Baseline FEV_1 _(mL)	High, middle, low	4	Burchfiel *et al. *[11]	Male	45-68	Mean	-24.8 (1.6)***	-16.1 (5.3)**	
						Diff	Base	-8.8 (5.6)	
									
Baseline FEV_1 _(mL/m^3^)	500+, 1-499	45	Townsend [73]	Male	35-57	Mean	28.1 (8.6)**	21.2 (15.6)	
						Diff	Base	6.9 (17.8)	
									
Baseline FEV_1_/FVC (%)	80+, 70-79, 1-69	5	Burrows *et al. *[10]	Male	20-70	Mean	27.9 (4.2)***		-3.4 (5.5)
						Diff	Base		31.3 (6.9)***
									
Obstruction at baseline	None, mild	13	Fletcher and Peto [31]	Male	30-59	Mean	24.8 (16.4)		7.0 (9.4)
						Diff	Base		17.8 (18.9)
									
Obstruction at baseline	None, mild	13	Fletcher *et al. *[6]	Male	30-59	Mean	22.1 (11.4)		4.0 (8.5)
						Diff	Base		18.1 (14.3)
									
Doctor visits for LRI^d^	0-0.24, 0.25-0.49, 0.50-0.99, 1.00-1.49, 1.50+	43	Kanner *et al. *[70]	Male	35-60	Mean	3.5 (0.8)***		2.9 (1.9)
						Diff	Base		0.7 (2.0)
									
Respiratory symptoms at baseline	No, Yes	29	Sherman *et al. *[53]	Male	25-74	Mean	4.6 (4.0)		4.3 (3.1)
						Diff	Base		0.3 (5.1)
									
Respiratory symptoms at baseline	No, Yes	29	Sherman *et al. *[53]	Female	25-74	Mean	1.9 (2.4)		-5.9 (5.0)
						Diff	Base		7.8 (5.5)
									
Bronchodilator responsiveness	No, Yes	34	Vollmer *et al. *[60]	Both	25-54	Mean	22.0 (30.1)	46.0 (22.7)	
						Diff	Base	-24.0 (37.7)	
									
Bronchodilator responsiveness	No, Yes	35	Vollmer *et al. *[60]	Both	25-54	Mean	10.0 (13.7)	14.0 (20.0)	
						Diff	Base	-4.0 (24.2)	
									
Histamine responsiveness	No, Yes	32	Rijcken *et al. *[5]	Male	25-54	Mean	3.2 (7.9)		4.5 (9.4)
						Diff	Base		-1.3 (12.3)
									
Histamine responsiveness	No, Yes	32	Rijcken *et al. *[5]	Female	25-54	Mean	5.8 (8.2)		5.9 (12.0)
						Diff	Base		-0.1 (14.5)
									
Occupational exposure	None, low, high	7	Sunyer *et al. *[24]	Male	20-44	Mean	0.8		-1.1
						Diff	Base		1.8
									
Occupational exposure	None, low, high	7	Sunyer *et al. *[24]	Female	20-44	Mean	1.2		6.2
						Diff	Base		-5.0
									
Exposure to gas and fumes	None, low, high	7	Sunyer *et al. *[24]	Male	20-44	Mean	1.5		-2.9
						Diff	Base		4.4
									
Exposure to gas and fumes	None, low, high	7	Sunyer *et al. *[24]	Females	20-44	Mean	-3.2		5.6
						Diff	Base		-8.9
									
Biological dust exposure	None, low, high	7	Sunyer *et al. *[24]	Males	20-44	Mean	0.2		2.5
						Diff	Base		-2.3
									
Biological dust exposure	None, low, high	7	Sunyer *et al. *[24]	Females	20-44	Mean	6.8		5.6
						Diff	Base		1.2
									
Mineral dust exposure	None, low, high	7	Sunyer *et al. *[24]	Males	20-44	Mean	0.0		2.3
						Diff	Base		-2.3
									
Mineral dust exposure	None, low, high	7	Sunyer *et al. *[24]	Females	20-44	Mean	2.0		6.5
						Diff	Base		-4.5

^a^FEV_1_, forced expiratory volume in 1 second; FVC, forced vital capacity. ^b^The trend is the estimated weighted increase in beta per level of exposure, except for study 7 where it is unweighted as no SEs are available. Trends are underlined if significant at *P *< 0.05. ^c^The difference in the trend compared to continuing smokers. Significant differences in trends are indicated by *P < 0.05, **P < 0.01 and ***P < 0.001. ^d^LRI, lower respiratory infection.

There is also evidence from study 43 that, in continuing smokers, beta increases with increasing number of doctor visits for lower respiratory infection. Though no such increase is seen in ex-smokers, the difference in trends is not statistically significant.

No clear evidence of an association with betas within or between smoking groups is evident in respect of respiratory symptoms at baseline (study 29), bronchodilator responsiveness (studies 34 and 35) or histamine responsiveness (study 32). The results from study 7 also do not suggest any marked relationship of beta to occupational exposure, though lack of SEs limits detailed interpretation.

## Discussion

A major objective of our review is to quantify and compare the rate of FEV_1 _decline (beta) in those who continue to smoke (continuing smokers) and those who give up smoking (quitters and ex-smokers). In an ideal world, this review would involve a number of large studies in which smoking habits, FEV_1 _levels and relevant confounding variables were measured at regular intervals and in which betas could be assessed separately for continuing smokers and for those who gave up, by time quit, on the basis of recently recorded smoking data. One could then distinguish between alternative possible models for FEV_1 _decline. For example, it might be that, following giving up smoking (and not subsequently restarting), the rate of decline in FEV_1 _drops immediately to a lower level than that of continuing smokers and continues at this level. Alternatively, it might be that, on giving up smoking, the rate of decline drops only slightly at first but then increases over time until it reaches a fixed level. The theory suggested by Fletcher and Peto [6] implies that the first situation may obtain, but there are few studies which present data in enough detail to distinguish such alternatives.

Anthonisen *et al. *[7], on the basis of a randomized clinical trial of smoking intervention (The Lung Health Study, study 43), did present a figure that suggests that giving up smoking leads to a reduced (and constant) beta quite quickly, though their study also suggests that in the first year or so after giving up, FEV_1 _levels may actually increase slightly. However, such data seem extremely rare, and the studies considered here include many in which FEV_1 _was recorded at only two time points, and some where, despite FEV_1 _being recorded at multiple time points, the data presented relate only to the average beta over the whole follow-up period. Studies where results are presented for more than one time period are relatively rare, and some of these studies do not adequately characterize smoking status at the beginning and end of each period studied. To allow assessment of FEV_1 _decline from a reasonable number of studies, therefore, we have summarized the data relating to the experience of a smoking group over a defined period, with the key information recorded being the smoking habits of that group at the beginning and end of the period and the beta estimated over the period studied. While the limitations of the available data mean that we cannot estimate the exact shape of the decline in FEV_1 _over time, our approach (which implicitly assumes a linear decline) is supported by the lack of relationship noted between beta and length of follow-up period (see Table 5).

Before discussing the results obtained, some other limitations of the data should be noted. Many of the 47 studies with relevant data are old, with 16 starting before 1970 and 42 beginning before 1990, and almost half of the studies provided data only for men. A number of the studies are quite small, with nine involving less than 100 individuals, implying very limited numbers in some of the smoking groups. In many of the studies, there was no adjustment for any variables, not even age or height. Smoking habits were not always defined at both the beginning and end of the time interval studied. FEV_1 _results were virtually never recorded after bronchodilator therapy as recommended for the diagnosis of COPD [8,9]. For many of the studies, estimates of the variability of the betas are not available, though for some estimates, the variability could be derived on the basis of SD estimates for other studies and knowledge of sample size. There are very limited data on how betas for a given smoking group vary by other factors of interest, as is evident from Table 7. It should also be pointed out that although there is a reasonable amount of information on how beta varies by amount smoked per day in continuing smokers (see Table 6), there are no such data for those who give up smoking. Also, comparisons of continuing smokers with quitters or ex-smokers are very often unadjusted for the amount smoked per day at the time when the quitters or ex-smokers were still smoking. We have not attempted to assess the individual studies for quality and susceptibility to bias, partly because there are no generally recognized methods for doing so for observational epidemiological studies, partly because a one-dimensional score for a study cannot really adequately summarize the multiple facets of its quality, and partly as differentially weighting (or rejecting) results from different studies based on an inevitably subjective score is always contentious, perhaps especially so when the study was supported by the tobacco industry.

Another possible limitation of our work concerns the completeness of our database, given the difficulty of being certain that all the relevant literature has been obtained, particularly when studies have been conducted over such a long period and given that some studies which clearly have the ability to provide relevant results seem never to have published findings in an appropriate format.

Despite all these limitations, we believe that the database assembled is of value in assessing the relationship of smoking habits, and particularly giving up smoking, to the magnitude of beta. A number of main conclusions can be drawn from our analyses.

First, beta in never smokers is clearly less than that in continuing smokers. The results summarized in Table 4 suggest that, whereas beta in continuing smokers is over 40 mL/yr, it is less than 30 mL/yr in never smokers. The difference exceeds 10 mL/yr and is highly significant (*P *< 0.001) in all the analyses shown. Though there is variation between blocks (that is, rows of Table 3) in the level of betas, the higher betas in continuing smokers is evident in virtually every block.

It is also clear that betas in ex-smokers, who gave up before the start of the period over which the FEV_1 _was measured, are quite similar to those in never smokers. In the inverse variance-weighted analyses adjusted for block, beta was estimated as 27.6 mL/yr, a nonsignificant 1.6 mL/yr lower than the estimate of 29.2 mL/yr for never smokers. Estimates for quitters (31.6 mL/yr for the same analyses) tend to be somewhat higher than for never smokers or ex-smokers, but are clearly lower than those in continuing smokers. Though variability in the estimates does not make the intermediate position of quitters well-defined, the results can plausibly be explained by the quitters having smoked for part of the period during which the betas were estimated. Data were not available to relate time of quitting to beta.

Our analyses also show that, in continuing smokers, there is a clear dose relationship with amount smoked, with an increase in beta of 0.33 mL/yr per cigarette/day. Though the data are relatively limited, they are consistent in showing a beta greater in the heaviest smokers than in the lightest smokers.

Four of the studies (4, 5, 13 and 45) provide information relating beta to smoking group by level of lung function, as determined by FEV_1_, FEV_1_/FVC or presence of mild obstruction. Studies 5, 13 and 45 present results which seem consistent with what has been termed the "horse-racing effect" [6,10], whereby reduced lung function predicts a rapid rate of decline simply because the rapid decline produced the reduced level of lung function in the first place. However, study 4 presents results which seem totally inconsistent with this finding, particularly in comparison with study 13. The results shown in Table 7 for this study are for the first 2 years follow-up, as SEs could not be derived for the full 6 years of follow-up. Though the strong tendency for betas to be higher in continuous smokers and quitters with a high baseline FEV_1 _seen in the first 2 years of follow-up seems not so marked for the full 6 years follow-up (see Table 5 of the source paper [11]), there is still no evidence of the horse-racing effect, as the authors note. Why this inconsistency is seen is not clear.

In all four studies, the trend in beta in relation to reduced lung function is weaker in quitters and ex-smokers than in continuing smokers. However, only in study 5, where a tendency for low baseline FEV_1_/FVC to predict an increased beta is clearly evident in continuing smokers but not evident in ex-smokers, is the difference from continuing smokers significant at *P *< 0.05.

The other factors considered in Table 7 (doctor visits for lower respiratory infection, bronchodilator responsiveness, histamine responsiveness and various aspects of occupational exposure) generally show no relationship with beta in continuing smokers, quitters or ex-smokers or with the difference in beta between continuing smokers and the other two smoking groups. The only exception was the significant tendency for beta in continuing smokers to increase with doctor visits. The evidence for each of these factors is very limited, each coming from a single study. While there do not appear to be other studies that allow assessment of differences in trends between continuing smokers and quitters or ex-smokers, it is possible that additional studies may provide evidence for the association in smokers or in the whole population, regardless of smoking habits. Because this review is mainly concerned with the study of effects of giving up smoking, we did not consider studies which did not report results for quitters or ex-smokers.

The same applies to the study characteristics considered in Table 5. Had we been specifically trying to answer the question whether beta varies by age or sex, much additional literature would have been considered. Of more interest is whether these study characteristics are related to the difference in betas between continuing smokers and quitters or ex-smokers. The main finding here is that the difference in betas between continuing smokers and quitters is greater where the estimates relate to individuals with specific respiratory diseases than when they relate to the general population. This is consistent with the theory that a susceptible proportion of smokers suffer a more rapid decline in lung function than do other smokers or those who have given up smoking. This susceptible proportion would be more likely both to have reduced lung function and be diagnosed with respiratory disease [6]. Other than having a greater beta, having reduced lung function, and being more likely to be diagnosed as having COPD, our review does not cast any light on characteristics linked to susceptibility in smokers.

For the purposes of designing a study comparing smokers and users of new-generation nicotine delivery products, it would be useful to know the level of decline in FEV_1 _one would expect over a defined time period in continuing smokers and those who give up smoking. Our analyses, presenting the results in terms of average FEV_1 _decline per year (beta) assume that the rate of decline is approximately constant over time, an assumption which is supported by the analyses presented in Table 5. Though this analysis is uncertain, being ecological in nature (as the relationship of beta to length of follow-up is evaluated only between studies), the strength of the association is clearly not strong. This suggests that our estimates of beta, based on 39 studies with an average follow-up period of 9 years, can be taken to apply both to short-term studies of say 5 years and to longer-term studies of, say, 15 years. It would seem reasonable to design a study comparing FEV_1 _declines in continuing smokers of conventional cigarettes and switchers to new products, assuming that beta reduces a somewhat conservative 10 mL/yr on quitting and that it reduces by perhaps 8 or 9 mL/yr in the switchers, provided that there is good toxicological evidence that these new products have little or no respiratory effect. For a 5-year study, we estimate that a comparison of continuing smokers and switchers would require about 120 smokers per group to have 80% power to detect a difference of 8 mL/yr at the *P *< 0.05 significance level, assuming participants do not change their smoking habits and ignoring dropouts. To detect a difference of 9 mL/yr would require about 95 smokers per group.

## Conclusions

While the available data have a number of limitations, it is possible to draw a number of conclusions relating to the annual rate of FEV_1 _loss (beta) in continuing smokers, quitters, ex-smokers and never smokers. Continuing smokers have a beta that is clearly greater, by more than 10 mL/yr, than in never smokers and is positively related to the number of cigarettes smoked per day. Betas in ex-smokers are similar to those in never smokers, and betas in quitters are only slightly greater. There is no clear evidence that differences in betas between continuing smokers and those who have given up smoking depend on age or sex, but differences are greater in studies of populations with respiratory disease than in general population studies. Some, but not all, studies suggest that betas are greater in those with reduced lung function, particularly in continuing smokers.

## Abbreviations

CI: confidence interval; COPD: chronic obstructive pulmonary disease; FEV_1_: forced expiratory volume in 1 second (mL/yr); FVC: forced vital capacity; *N*: number of subjects; SD: standard deviation; SE: standard error.

## Competing interests

PNL, founder of P.N. Lee Statistics and Computing Ltd., is an independent consultant in statistics and an adviser in the fields of epidemiology and toxicology to a number of tobacco, pharmaceutical and chemical companies. JSF works for P.N. Lee Statistics and Computing Ltd.

## Authors' contributions

PNL carried out the literature search and, with JSF's assistance, extracted the relevant data, carried out the statistical analyses, prepared a detailed report and drafted the manuscript for publication.

## Pre-publication history

The pre-publication history for this paper can be accessed here:

http://www.biomedcentral.com/1741-7015/8/84/prepub

## Supplementary Material

Additional file 1 **Detailed search strategy**. Fuller details of the search, summarized in Figure 1 in the paper, including the stages at which each of the examined papers was accepted and rejected. Note that the reference list of the paper itself includes not only those references cited in the paper but also those cited in this additional file. Click here for file

Additional file 2 **Data recorded on the study and beta databases**. Fuller details of the variables recorded on the databases as summarized in Methods: Data entry. Click here for file
