# Supplementary material for: Systematic review of the evidence relating FEV1 decline to giving up smoking
Source: BMC Med. 2010 Dec 14;8:84. doi: 10.1186/1741-7015-8-84 (PMC3017006; doi:10.1186/1741-7015-8-84)
Supplement: Additional file 1 — Detailed search strategy. Fuller details of the search, summarized in Figure 1 in the paper, including the stages at which each of the examined papers was accepted and rejected. Note that the reference list of the paper itself includes not only those references cited in the paper but also those cited in this additional file. [file 1741-7015-8-84-S1.DOC]

“Systematic review of the evidence relating

FEV_1_ decline to giving up smoking”

Detailed search strategy

Authors : P N Lee and J S Fry

Date : 21^st^ July 2010

1. **Searches carried out in 2009**

1.1 Stage 1: Publications obtained from a MedLine search

On 6^th^ April 2009 a MedLine search was conducted on “(Lung function or FEV_1_ or decline in FEV_1_) and (exsmokers or smoking cessation)” limited to “Humans” and “to All adults: 19+ years)”. There was no restriction on date or language. This identified 514 papers. Abstracts were printed out and examined to determine whether papers were of possible relevance. 99 papers were selected and copies obtained of the papers. 34 of these papers were accepted and 65 rejected. Table 1 gives fuller details, showing the references of the 99 papers, the 20 study numbers the accepted papers related to, and the reasons for rejection.

1.2 Stage 2: Publications cited in review by Willemse *et al* [3]

Tables 2 and 3 of this review cite results for FEV_1_ decline in smokers, exsmokers, quitters and nonsmokers, with Table 2 giving results for those without chronic respiratory symptoms and Table 3 giving results for those with chronic bronchitis or COPD. These references and also those given in the text of the relevant sections of the paper were examined. 11 additional papers were identified and copies obtained. 9 were accepted and 2 rejected. See Table 2 for details.

1.3 Stage 3: Publications cited in IARC review [1]

The IARC handbook “Reversal of risk after quitting smoking” [1] includes an unnumbered chapter entitled “Change in risk of chronic obstructive pulmonary disease after smoking cessation”. Relevant sections of this and also of another chapter, “The effects of smoking cessation on chronic lung diseases in China”, were examined for additional references. 14 were identified, with 3 accepted and 22 rejected. See Table 3 for details.

1.4 Stage 4: Publications identified in a literature review by Thornton

Some years before, Alison Thornton, a colleague, had carried out a very detailed literature review of the relationship between smoking and various aspects of respiratory disease, including FEV_1_ loss. Although this work was never completed, the files for FEV_1_ (obtainable on request) were examined for additional references. 16 were identified, with 6 accepted and 10 rejected. See Table 4 for details.

1.5 Publications obtained from references cited in accepted papers

By the end of stages 1 to 4 a total of 52 papers had been accepted. The literature cited in these papers (and also in relevant review papers identified) was then studied and a total of 103 additional references were investigated. 43 produced accepted papers, and 60 rejected papers. See Table 5 for details.

2. **Updated searches carried out in 2010**

2.1 Stage 6: Publications obtained from an updated MedLine search

At the request of the journal the MedLine search conducted on 6^th^ April 2009 was repeated on 6^th^ July 2010, including all publications to 30^th^ June 2010. Although the conditions of the search were identical and the period covered longer, the total numbers of papers identified, 461, was less than identified in 2009. No clear explanation for this could be found and the abstracts of all 461 papers were examined. Of the 99 papers selected for further consideration in 2009, 96 appeared in the 2010 search, the other 3 [77-79] all having been rejected earlier. A further 8 papers were identified as of possible relevance, 1 of which was accepted and 7 were rejected. See Table 6 for details.

2.2 Stage 7: Publications obtained from references cited in accepted papers

References in the paper accepted at Stage 6 [62] were examined and a further 9 publications were examined. All were rejected. See Table 7 for details.

3. **Accumulation of studies**

3.1 Table 8 gives details of the stages at which the final set of studies analyzed were discovered. Of the total of 47, 20 were identified in the original MedLine search in 2009, with a further 6 identified from the review by Willemse *et al* [3] and a further 5 from the unpublished literature review by Alison Thornton. A further 15 studies came from references cited in the papers identified, with 1 new additional study identified in the updated search in 2010.

4. **Summary**

4.1 Flow diagram

Figure 1 summarizes the whole search process.

TABLE 1

Stage 1: Using the first MedLine search in 2009

| **Accepted papers** |  |  |
| --- | --- | --- |
|  |  |  |
| Final study number | No of papers | References |
|  |  |  |
| 1 | 1 | [12] |
| 3 | 1 | [16] |
| 4 | 1 | [11] |
| 5 | 4 | [10,21,80,81] |
| 7 | 1 | [23] |
| 10 | 1 | [27] |
| 15 | 1 | [33] |
| 18 | 1 | [36] |
| 19 | 1 | [37] |
| 22 | 1 | [42] |
| 24 | 1 | [48] |
| 26 | 2 | [50,82] |
| 28 | 1 | [52] |
| 29 | 1 | [54] |
| 30 | 1 | [55] |
| 31 | 1 | [56] |
| 32 | 1 | [4] |
| 43 | 10 | [7,69-71,83-88] |
| 44 | 1 | [72] |
| 45 | 2 | [74,89] |
|  |  |  |
| Total | 34 |  |
|  |  |  |
| **Rejected papers** |  |  |
|  |  |  |
| Reason for rejection | No of papers | Reference |
|  |  |  |
| Alpha-1-AT deficient subjects | 1 | [90] |
| Data for FEV_0.75_ only | 3 | [91-93] |
| Chronic spinal cord injury patients | 1 | [94] |
| Follow-up too short | 10 | [95-104] |
| No follow-up data | 2 | [105,106] |
| No relevant data in quitters | 39 | [77-79,107-142] |
| Not a prospective study | 3 | [143-145] |
| Review paper | 2 | [3,146] |
| Asbestos-exposed subjects | 1 | [147] |
| Steelworkers | 2 | [148,149] |
| Study of children | 1 | [150] |
|  |  |  |
| Total | 65 |  |

TABLE 2

Stage 2: Using review by Willemse *et al* [3]

| **Accepted papers** |  |  |
| --- | --- | --- |
|  |  |  |
| Final study number | No of papers | References |
|  |  |  |
| 5 | 1 | [151] |
| 9 | 1 | [26] |
| 13 | 1 | [31] |
| 21 | 2 | [41,151] |
| 23 | 1 | [44] |
| 29 | 1 | [53] |
| 41 | 1 | [67] |
| 42 | 1 | [68] |
| 43 | 1 | [152] |
|  |  |  |
| Total | 9* |  |
|  |  |  |
| **Rejected papers** |  |  |
|  |  |  |
| Reason for rejection | No of papers | Reference |
|  |  |  |
| No relevant data in quitters | 1 | [153] |
| Follow-up too short | 1 | [154] |
|  |  |  |
| Total | 2 |  |
|  |  |  |
| * One paper [151] described results for both study 5 and study 21 | | |

TABLE 3

Stage 3: Using IARC review [1]

| **Accepted papers** |  |  |
| --- | --- | --- |
|  |  |  |
| Final study number | No of papers | References |
|  |  |  |
| 5 | 1 | [155] |
| 7 | 1 | [24] |
| 23 | 1 | [46] |
|  |  |  |
| Total | 3 |  |
|  |  |  |
| **Rejected papers** |  |  |
|  |  |  |
| Reason for rejection | No of papers | Reference |
|  |  |  |
| Follow-up too short | 4 | [156-159] |
| No relevant data in quitters | 1 | [160] |
| Not a prospective study | 1 | [161] |
| Paper unobtainable | 4 | [162-165] |
| Review | 1 | [166] |
|  |  |  |
| Total | 11 |  |
|  |  |  |

TABLE 4

Stage 4: Using a literature review by Thornton

| **Accepted papers** |  |  |
| --- | --- | --- |
|  |  |  |
| Final study number | No of papers | References |
|  |  |  |
| 2 | 1 | [14] |
| 3 | 1 | [15] |
| 6 | 1 | [22] |
| 8 | 1 | [25] |
| 37 | 1 | [63] |
| 38 | 1 | [64] |
|  |  |  |
| Total | 6 |  |
|  |  |  |
| **Rejected papers** |  |  |
|  |  |  |
| Reason for rejection | No of papers | Reference |
|  |  |  |
| No relevant data in quitters | 5 | [167-171] |
| Not a prospective study | 2 | [172,173] |
| Cotton mill workers | 1 | [174] |
| Exposed to toluene diisocyanate | 1 | [175] |
| Exposed to volcanic ash | 1 | [176] |
|  |  |  |
| Total | 10 |  |
|  |  |  |

TABLE 5

Stage 5: Using references cited in accepted papers in 2009

| **Accepted papers** |  |  |
| --- | --- | --- |
|  |  |  |
| Final study number | No of papers | References |
|  |  |  |
| 1 | 1 | [13] |
| 3 | 6 | [17-20,177,178] |
| 5 | 3 | [179-181] |
| 11 | 1 | [28] |
| 12 | 2 | [29,182] |
| 13 | 2 | [6,30] |
| 14 | 1 | [32] |
| 16 | 1 | [34] |
| 17 | 1 | [35] |
| 20 | 3 | [38-40] |
| 21 | 3 | [183-185] |
| 22 | 2 | [43,186] |
| 23 | 3 | [45,47,187] |
| 25 | 1 | [49] |
| 27 | 1 | [51] |
| 31 | 1 | [57] |
| 32 | 2 | [5,58] |
| 33 | 1 | [59] |
| 34 | 1 | [60] |
| 35 | 1 | [60] |
| 36 | 1 | [61] |
| 39 | 1 | [65] |
| 40 | 1 | [66] |
| 43 | 1 | [188] |
| 45 | 2 | [73,189] |
| 46 | 1 | [75] |
|  |  |  |
| Total | 43* |  |
|  |  |  |
| **Rejected papers** |  |  |
|  |  |  |
| Reason for rejection | No of papers | Reference |
|  |  |  |
| Alpha-a-AT deficient subjects | 5 | [190-194] |
| Data for FEV_0.75_ only | 2 | [195,196] |
| Subjects with abnormal static lung compliance | 1 | [197] |
| Subjects with carcinoembryonic antigen abnormality | 1 | [198] |
| Follow-up too short | 3 | [199-201] |
| No follow-up data | 2 | [202,203] |
| No relevant data in quitters | 30 | [204-233] |
| Not a prospective study | 8 | [234-241] |
| Review paper | 2 | [242,243] |
| Ash exposed loggers | 1 | [244] |
| Grain elevator workers | 1 | [245] |
| Iron miners | 2 | [246,247] |
| Pulp mill workers | 1 | [226] |
| Steelworkers | 1 | [248] |
|  |  |  |
| Total | 60 |  |
|  |  |  |
| * One paper [60] described results for both study 34 and study 35 | | |

TABLE 6

Stage 6: Using the second MedLine search in 2010

| **Accepted papers** |  |  |
| --- | --- | --- |
|  |  |  |
| Final study number | No of papers | References |
|  |  |  |
| 47 | 1 | [62] |
|  |  |  |
| Total | 1 |  |
|  |  |  |
| **Rejected papers** |  |  |
|  |  |  |
| Reason for rejection | No of papers | Reference |
|  |  |  |
| No relevant data in quitters | 6 | [249-254] |
| Review paper | 1 | [255] |
|  |  |  |
| Total | 7 |  |

TABLE 7

Stage 7: Using references cited in the additional paper in 2010

| **Accepted papers** |  |  |
| --- | --- | --- |
|  |  |  |
| Final study number | No of papers | References |
|  |  |  |
| None |  |  |
|  |  |  |
| **Rejected papers** |  |  |
|  |  |  |
| Reason for rejection | No of papers | Reference |
|  |  |  |
| No relevant data in quitters | 8 | [256-263] |
| Review paper | 1 | [264] |
|  |  |  |
| Total | 9 |  |

TABLE 8

Stage at which the 47 studies were first identified

| Study number | Stage | Study number | Stage |
| --- | --- | --- | --- |
|  |  |  |  |
| 1 | 1 | 27 | 5 |
| 2 | 4 | 28 | 1 |
| 3 | 1 | 29 | 1 |
| 4 | 1 | 30 | 1 |
| 5 | 1 | 31 | 1 |
| 6 | 4 | 32 | 1 |
| 7 | 1 | 33 | 5 |
| 8 | 4 | 34 | 5 |
| 9 | 2 | 35 | 5 |
| 10 | 1 | 36 | 5 |
| 11 | 5 | 37 | 4 |
| 12 | 5 | 38 | 4 |
| 13 | 2 | 39 | 5 |
| 14 | 5 | 40 | 5 |
| 15 | 1 | 41 | 2 |
| 16 | 5 | 42 | 2 |
| 17 | 5 | 43 | 1 |
| 18 | 1 | 44 | 1 |
| 19 | 1 | 45 | 1 |
| 20 | 5 | 46 | 5 |
| 21 | 2 | 47 | 6 |
| 22 | 1 |  |  |
| 23 | 2 |  |  |
| 24 | 1 | Total studies | Stage |
| 25 | 5 | 20 | 1 |
| 26 | 1 | 6 | 2 |
|  |  | 0 | 3 |
|  |  | 5 | 4 |
|  |  | 15 | 5 |
|  |  | 1 | 6 |

FIGURE 1: Flow diagram

Medline search

April 6^th^ 2009

514 papers identified

415 rejected from examination of abstracts

99papers examined

34papers accepted, describing 20 studies

65papers rejected (Reason 2: 2, R3: 3, R4 1, R5: 2, R6: 3, R7: 10, R8: 2, R9: 3, R10 39)

1.

11new papers examined

9new papers accepted

6 new studies identified

2new papers rejected (R7: 1, R10: 1)

From review by

Willemse *et al* (2004)

2.

From review by

IARC (2007)

14new papers examined

3new papers accepted

0new studies identified

11new papers rejected (R1: 4, R5: 1, R6: 1, R7: 4, R10: 1)

4.

From literature review

by Thornton

16new papers examined

6new papers accepted

5new studies identified

10new papers rejected (R3: 3, R6: 2, R10: 5)

5.

From secondary

references of

accepted papers

103new papers examined

43new papers accepted

15new studies identified

60new papers rejected (R2: 7, R3: 6, R5: 2, R6: 8, R7: 3, R8: 2, R9: 2, R10: 30)

3.

6.

Updating MedLine search

to June 30^th^ 2010

8new papers examined

1new paper accepted

1new study identified

7 new papers rejected (R5: 1, R10: 6)

Further secondary references of newly accepted papers

9new papers examined

0new papers accepted

0new studies identified

9new papers rejected (R5: 1, R10: 8)

Overall summary

7.

260papers examined

96papers accepted

47studies identified

164papers rejected:

R1: 4Unobtainable

R2: 9Patients with specified conditions not COPD

R3: 12Workers in high risk occupations

R4: 1Study of children

R5: 7Review, no new studies mentioned

R6: 14Not a prospective study

R7: 18Follow-up too short

R8: 4No follow-up data

R9: 5Only data for FEV_0.75_

R10: 90No relevant data on FEV_1_ decline in those who give up smoking

7.
