# Supplementary material for: Systematic review of the evidence relating FEV1 decline to giving up smoking
Source: BMC Med. 2010 Dec 14;8:84. doi: 10.1186/1741-7015-8-84 (PMC3017006; doi:10.1186/1741-7015-8-84)
Supplement: Additional file 2 — Data recorded on the study and beta databases. Fuller details of the variables recorded on the databases as summarized in Methods: Data entry. [file 1741-7015-8-84-S2.DOC]

“Systematic review of the evidence relating

FEV_1_ decline to giving up smoking”

Data recorded on the study and beta databases

Authors : P N Lee and J S Fry

Date : July 2010

**STUDY DATABASE** (1 record per study, each study being identified by a 6 character reference code)

| Section | Field | Further details |
| --- | --- | --- |
| **Study description** |  |  |
|  | First author |  |
|  | Study short name |  |
|  | Full study title |  |
|  | Study sex | 1 = both, 2 = male, 3 = female. |
|  | Lowest age in study |  |
|  | Highest age in study (at baseline) |  |
|  | Highest age in study at final follow up |  |
|  | Region | 1 = USA, 2 = Canada, 3 = S/C America, 4 = UK, 5 = Western Europe, 6 = Scandinavia, 7 = E Europe, 8 = SE Europe/Balkans, 9 = Middle East/S Asia, 10 = SE Asia/Pacific, 11 = Far East, 12 = Australia/NZ, 13 = Africa, 14 = Multicountry. |
|  | Country and location with country |  |
|  | Start year of study |  |
|  | End year of study | Refers to baseline. |
|  | Final follow up year |  |
|  | Principal publication year |  |
|  | Reference ID of principal publication | Which is the principal publication is sometimes somewhat arbitrary. |
|  | Reference ID of additional publication(s) |  |
|  | Study status for FEV_1_ project |  |
|  | Comments | In each section, free text comments, linked if appropriate to the relevant field, allow further data description. |
|  |  |  |
| **Study design** |  |  |
|  | Study type | 1 = prospective, 2 = intervention. |
|  | Type of population | 1 = all (general population), 2 = COPD patients, 3 = CB patients, 4 = Emphysema patients, 5 = other health based criteria. |
|  | Population type | Describe nature of population in detail. |
|  | Medical exclusions |  |
|  | Other exclusions |  |
|  | Bronchodilator used for FEV_1_ | 1 = results entered after use, 2 = mentioned, but no useful results, 3 = not mentioned. |
|  | Total number of subjects | Total entering follow-up even if not used in analysis. |
|  | Comments |  |
|  |  |  |
| **Results for FEV_1_** |  | For all six fields enter 1 if results are available. |
|  | FEV_1_ decline per year |  |
|  | FEV_1_ decline per year relative to never smokers |  |
|  | FEV_1_ decline per year relative to continuing smokers |  |
|  | FEV_1_ decline per year divided by height cubed | Data not entered on beta database. |
|  | FEV_1_ change as % of predicted | Data not entered on beta database. |
|  | Other indices of FEV1 | Data not entered on beta database. |
|  | Comments |  |
|  |  |  |
| **Confounders used for FEV_1_ decline** |  | Only relates to results actually entered on the beta database. For most fields enter the number of relevant confounders. |
|  | Total number of adjustment factors used |  |
|  | Adjusted for sex |  |
|  | Adjusted for age |  |
|  | Adjusted for race |  |
|  | Adjusted for location within study |  |
|  | Adjusted for aspects of study design |  |
|  | Adjusted for SES (inc education) |  |
|  | Adjusted for occupation |  |
|  | Adjusted for height |  |
|  | Adjusted for weight |  |
|  | Adjusted for baseline lung function |  |
|  | Adjusted for other aspects of smoking |  |
|  | Adjusted for other factors |  |
|  | Comments |  |
|  |  |  |
| **Smoking results available** |  | Only relates to results actually entered on the beta database. For each field enter 1 if result entered. |
|  | For continuing smokers |  |
|  | For ex smokers |  |
|  | For quitters in follow-up |  |
|  | For never smokers |  |
|  | For continuing smokers (relative to never smokers) |  |
|  | For ex-smokers (relative to never smokers) |  |
|  | For quitters (relative to never smokers) |  |
|  | For quitters (relative to continuing smokers) |  |
|  | By amount smoked |  |
|  | By duration of quitting |  |
|  | By pack-years |  |
|  | By duration of smoking |  |
|  | By age started smoking |  |
|  | For other aspects of smoking |  |
|  | Comments |  |
|  |  |  |
| **Stratified by other factors** |  | Only relates to results actually entered on the beta database. For each field enter 1 if result entered. |
|  | Age |  |
|  | Sex |  |
|  | Race |  |
|  | Location |  |
|  | Baseline lung function |  |
|  | Other factors of particular subset |  |
|  | Comments |  |
|  |  |  |
| **Values available** |  |  |
|  | Number of records on beta database |  |

**BETA DATABASE** (1 record per beta, each beta being identified by the study reference code and the number of the estimate within study)

| Section | Field | Further details |
| --- | --- | --- |
| **Beta description** |  |  |
|  | Number of beta estimate within study | This usually starts from 1 and is consecutive. Values must be unique within a study. |
|  | Smoking status at start of period | 1 = never, 2 = ex, 3 = current, 4 = ever, 5 = non (i.e. never + ex). Entered as unknown in cases where the subjects were known to be quitters at the end of the period and where the smoking status at the start was unknown. Never smokers include never cigarettes in some studies, but the definition of never smokers is usually unclear. |
|  | Smoking product at start of period | 1 = any, 2 = cigarettes ($\pm$ other), 3 = cigarettes only, 4 = pipes or cigars only, 5 = pipe only. Entered only for those who ever smoked |
|  | Cigarette type at start of period | 1 = any, 2 = manufactured ($\pm$ handrolled), 3 = manufactured only. Entered only if cigarette smoker. |
|  | Dose response at start of period | 1 = all, 2 = level 1, 3 = level 2, 4 = level 3, 5 = level 4. Only entered for smoking status at start = 2, 3 or 4. Level 1 means that the data are for the whole group and not a specific dose level. Levels 2-5 relate to successive levels of a defined dose-response relationship. This dose-response relationship should be further defined either by the next field if it represents successive levels of a non-measured variable (e.g. low tar, high tar) or by the subsequent three fields for a measured variable. |
|  | Description of dose at start of period | A character field describing the level of a non-measured field. Only entered if it relates to a dose-response relationship. |
|  | Measure of exposure at start of period | c = cigarettes per day, p = pack-years, y = years smoked, g = grams of tobacco, f = low v high tar, b = filter v plain, v = change in cigs/day. |
|  | Low value at start of period |  |
|  | High value at start of period |  |
|  | Smoking status at end of period | Levels as for smoking status at start of period, plus 6 = intermittent quitter. Note that details on smoking habits at the end of the period are often incomplete. |
|  | Smoking product at end of period | Further details of the end of period fields are the same as for the corresponding start of period fields. |
|  | Cigarette type at end of period |  |
|  | Dose response at end of period |  |
|  | Description of dose at end of period |  |
|  | Measure of exposure at end of period |  |
|  | Low value at end of period |  |
|  | High value at end of period |  |
|  | Source | The publication and page/ table number. |
|  | Comments |  |
|  |  |  |
| **Stratifying variable** |  | The first three fields are always entered. The rest only apply where the result is for a particular stratum. |
|  | Sex | 1 = both, 2 = male, 3 = female. |
|  | Lowest age of subjects studied |  |
|  | Highest age of subjects studied |  |
|  | Stratifying variables | 1 = chronic mucous hypertension, 2 = occupational exposure, 3 = IgE level (IU/ml), 4 = respiratory symptoms at base, 5 = alpha-1 antitrypsin deficiency, 6 = wheal size (mm), 7 = alcohol (oz/wk), 8 = doctor visits for lower respiratory infection, 9 = bronchodilator responsiveness, 10 = baseline FEV_1_ level (c1/m3), 11 = exposure to gas and fumes, 12 = study centre, 13 = biological dust, exposure, 14 = mineral dust exposure, 15 = obstruction, 16 = baseline FEV_1_/FVC, 17 = race, 18 = baseline FEV_1_ level (ml), 19 = pulmonary impairment, 20 = baseline FEV_1_ level (ml/m^3^), 21 = histamine responsiveness. |
|  | Level of stratifying variable | 1 is entered only when the result relates to a subset of the population, with results not available for other subsets. 2, 3… are entered to describe successive levels of a stratifying variable. |
|  | Description of non-numeric level |  |
|  | Low value | For numeric levels |
|  | High value | For numeric levels |
|  | Intervention group | 1 = treatment 1, 2 = treatment 2, 3 = treatment 3, 4 = Treatment 4, 5 = Treatments 1+2, 6 = Treatments 1+2+3, 7 = Treatments 2+3. This describes which intervention study groups the Beta relates to. |
|  | Intervention treatment description | 1=UC (no intervention), 2 = SIP (smoking intervention, placebo inhaler), 3 = SIA (smoking intervention, bronchodilator), 4 = UC+SIP (no intervention + smoking intervention, placebo inhaler), 5 = control + smoking intervention, 6 = UC+SIP+SIA (any intervention, any inhaler), 7 = SIP+SIA (smoking intervention, any inhaler), 8 = UC+SI (usual care + special intervention). |
|  | Comments |  |
|  |  |  |
| **Beta adjustment** |  |  |
|  | Adjusted for sex | Enter 1 if adjustment made. |
|  | Adjusted for age | Enter 1 if adjustment made. |
|  | Adjusted for other aspects of smoking | Enter number of adjustment variables. |
|  | Adjusted for baseline FEV_1_ | Enter 1 if adjustment made. |
|  | Adjusted for other variables | Enter number of adjustment variables. |
|  | Post-bronchodilator measurement | This is an indicator whether or not the FEV_1_ measurement was taken after use of a bronchodilator. |
|  | Comments |  |
|  |  |  |
| **Beta data** |  |  |
|  | Type of beta | 1 = direct, 2 = relative to never smokers, 3 = relative to current smokers. |
|  | Beta values | Positive values indicate declines. |
|  | Lower 95% CI |  |
|  | Upper 95% CI |  |
|  | Standard deviation |  |
|  | Standard error |  |
|  | Number of subjects beta based on |  |
|  | Unexposed group | If beta relative to never smokers, 1 = never anything, 2 = never cigarettes.If beta relative to current smokers, 1 = continuing anything, 2 = continuing cigarettes. |
|  | Derivation | 1 = data as given in source, 2 = Beta and/or SD estimatedfrom numbers, 3 = data read from graph, 4 = Beta original, SD from extrapolation, 5 = Beta, SD/SE original, N estimated. |
|  | Length of period studied (years) | Often an approximation. |
|  | Comments |  |
